# Effect of Photo-Mediated Ultrasound Therapy on Nitric Oxide and Prostacyclin from Endothelial Cells

**DOI:** 10.3390/app12052617

**Published:** 2022-03-03

**Authors:** Madhumithra Subramanian Karthikesh, Sa Wu, Rohit Singh, Yannis Paulus, Xueding Wang, Xinmai Yang

**Affiliations:** 1 Bioengineering Graduate Program, University of Kansas, Lawrence, KS 66045, USA;; 2 Institute for Bioengineering Research, University of Kansas, Lawrence, KS 66045, USA;; 3 Department of Mechanical Engineering, University of Kansas, Lawrence, KS 66045, USA; 4 Department of Biomedical Engineering, University of Michigan, Ann Arbor, MI 48109, USA;; 5 Department of Ophthalmology and Visual Sciences, University of Michigan, Ann Arbor, MI 48109, USA

**Keywords:** ultrasound, photo-mediated ultrasound therapy, cavitation, endothelial cells, nitric oxide, blood vessel model

## Abstract

Several studies have investigated the effect of photo-mediated ultrasound therapy (PUT) on the treatment of neovascularization. This study explores the impact of PUT on the release of the vasoactive agents nitric oxide (NO) and prostacyclin (PGI_2_) from the endothelial cells in an in vitro blood vessel model. In this study, an in vitro vessel model containing RF/6A chorioretinal endothelial cells was used. The vessels were treated with ultrasound-only (0.5, 1.0, 1.5 and 2.0 MPa peak negative pressure at 0.5 MHz with 10% duty cycle), laser-only (5, 10, 15 and 20 mJ/cm^2^ at 532 nm with a pulse width of 5 ns), and synchronized laser and ultrasound (PUT) treatments. Passive cavitation detection was used to determine the cavitation activities during treatment. The levels of NO and PGI_2_ generally increased when the applied ultrasound pressure and laser fluence were low. The increases in NO and PGI_2_ levels were significantly reduced by 37.2% and 42.7%, respectively, from 0.5 to 1.5 MPa when only ultrasound was applied. The increase in NO was significantly reduced by 89.5% from 5 to 20 mJ/cm^2^, when only the laser was used. In the PUT group, for 10 mJ/cm^2^ laser fluence, the release of NO decreased by 76.8% from 0.1 to 1 MPa ultrasound pressure. For 0.5 MPa ultrasound pressure in the PUT group, the release of PGI_2_ started to decrease by 144% from 15 to 20 mJ/cm^2^ laser fluence. The decreases in NO and PGI_2_ levels coincided with the increased cavitation activities in each group. In conclusion, PUT can induce a significant reduction in the release of NO and PGI_2_ in comparison with ultrasound-only and laser-only treatments.

## 1. Introduction

Photo-mediated ultrasound therapy (PUT) is a non-invasive technique based on photoacoustic cavitation produced by applying ultrasound bursts and laser pulses synchronously [1–4]. The strong optical absorption of hemoglobin enables the selective treatment of blood vessels. PUT has been shown to cause minimal collateral damage to adjacent tissues [5] with weak optical absorptions. PUT was also shown to be highly precise in targeting the blood vessels and efficient compared to laser-only treatment [6]. The clinical potential of PUT is currently explored for treating neovascularization and vascularization in the choroid and retina, which lead to the pathogenesis of age-related macular degeneration and diabetic retinopathy [7,8].

The main mechanism of PUT is enhanced cavitation activity in the targeted vessels [9–16]. The enhanced cavitation activity can induce strong mechanical stresses in the vessels. These stresses directly influence the physiological functions of blood vessels by impacting platelets, red blood cells, and endothelial cells lining the inner walls of blood vessels [9,10], resulting in coagulation [13], hemorrhage [12,17], and vasoconstriction. To date, the impact of PUT on endothelial cells has not been investigated.

Vascular endothelial cells play important roles in many physiological functions of blood vessels [18,19]. Both laser treatment and ultrasound can impact endothelial functions. The effect of ultrasound on vascular endothelial cells has been examined for its applications in drug delivery [20–25], gene therapy [24,26–28], wound healing [29], deoxyribonucleic acid and protein synthesis [30], tumor dissection [31], and cancer therapies [32–34]. The effect of laser treatment on vascular endothelial cells has been investigated in wound repair [35,36], tissue regeneration [37], and therapeutic angiogenesis [38].

Nitric oxide (NO) regulates vascular, biological, neurological, and immunological functions [39,40]. It can be released when the endothelium is subjected to shear stress due to blood flow and changes related to blood flow [41–44]. Prostacyclin (PGI_2_) is co-released with NO from the endothelial cells and forms a specific association in the regulation of vascular and platelet function [45]. Shear stress has been found to increase the release of PGI_2_ from the endothelial cells [46].

We developed a blood vessel model based on the polydimethylsiloxane (PDMS) channel. PDMS has been widely used in developing blood vessel models in vitro for various purposes, such as studies of tumor microenvironment, endothelial biochemical production, drug transport, and blood–brain barriers [47–51]. PDMS is safe, biocompatible, hemocompatible, elastic, clear, and stable [52–58]. Its biologically inert surface prevents microbial growth, making it ideal for biomedical applications. It is also porous and extremely permeable to gases such as oxygen and carbon dioxide. This makes it perfect for cultivating cells for in vitro studies and hydrogels for wound healing and contact lens [59,60]. To promote cellular adhesion to the channel, the channel is coated with type I collagen [61], fibrin [48,62], or fibronectin [47,49–51]. Collagen is used in this study as it constitutes the major structural protein in the body [63]. To facilitate endothelial blood vessel formation, type I collagen is used [48]. Following this, the cell is seeded and allowed to reach confluence. Though micro-circular channels fabricated using various combinations of microfabrication techniques were reported [64,65], none of them were specifically designed for ultrasound-related testing. There is no evidence of any study that uses a similar in vitro vessel model to understand the effect of ultrasound, laser treatment and PUT on endothelial cells. We used needles as molds, as used by Chrobak et al. [62] and Sfriso et al. [49], to produce a circular vessel model with no irregularities. The previous in vitro studies involved the treatment of endothelial cells grown on a Petri dish or well plates. The cellular effects in these cases are not completely like the in vivo effects, as the cell culture is in 2D, and the impact of shear stress induced due to the circular cross-section and flow is eliminated. As a vessel model mimics the blood vessel in vitro, the in vitro effects are expected to be more comparable to the in vivo effects.

PUT has been shown to shut down the blood vessels in the neovascularization-induced rabbit eye [4] by possibly stimulating endothelial cells to achieve the desired bioeffect based on the enhanced cavitation activity [66–68]. The motivation behind this research is to understand the changes in the levels of PGI_2_ and NO released with the endothelial cells in the choroidal blood vessels during PUT. Therefore, we used chorioretinal endothelial cells in the in vitro model. The cells were cultured under static conditions. The static culture does not cause any mechanotransduction associated with shear stress on the endothelial cells, which may result in cellular changes involving the release of any biochemicals or change in the expression of the markers [69]. This study provides an understanding of the release of PGI_2_ and NO when treating with ultrasound-only, laser-only, and PUT.

## 2. Materials and Methods

### 2.1. In Vitro Circular Cross-Section Microchannel

PDMS (Sylgard^^™^^ 184, Dow Corning, Wiesbaden, Germany) scaffold was prepared by mixing elastomer silicone and curing agent in a 1:10 ratio. The PDMS mixture was then degassed in a vacuum oven (NAPCO, Thermo Scientific, Waltham, MA, USA) to eliminate the bubbles that arose due to the stirring involved with the mixing of the elastomer and curing agent. The mixture was then poured into standard cuvettes (14955125, Fisherbrand^^™^^, Pittsburgh, PA, USA). The circular cross-section microchannel was made in this scaffold by inserting a 27G 1–1/2” needle (BD Sciences, Franklin Lakes, NJ, USA) in the center of the cuvette. The needle was held in the center using tape. The PDMS with the needles in the cuvette was cured overnight at room temperature to obtain the channel mimicking the blood vessel. The PDMS scaffold containing the channel was then released by removing the cuvette. The ends of the channels were cut so that the channel was open-ended to facilitate the exchange of gases during cell seeding. The microchannel mimicked blood vessels with a radius of 450 μm and a length of 3.5 cm. A picture of the fabricated microchannel is shown in Figure S1. The PDMS scaffold was sterilized in 70% ethanol for 30 min before collagen coating. The high-concentration rat tail type I collagen (354249, Corning, AZ, USA) of 350 μg/mL was used for coating these channels. The collagen was injected into the channels using a sterile syringe (BD Sciences). The channel was then incubated at 37 C for 1 h. The excess collagen was aspirated by drying the channel for two hours in a fume hood.

To mimic the choroidal vasculature of the eye, *Macaca mulatta* chorioretinal endothelial cells RF/6A (ATCC^®^ CRL-1780^™^) were used. The cells were cultured in a 75 cm^2^ flask placed in a cell culture incubator (Nunc ^™^, Thermo Scientific, Waltham, MA, USA) at 37°C and 5% CO_2_. The cells were split in a 1:2 ratio as they reached 80% confluence. Eagle’s minimum essential medium (EMEM) (ATCC^®^ 30–2003^™^) culture media supplemented with 10% fetal bovine serum (ATCC^®^ 30–2020^™^) and 100 IU/mL penicillin and 100 μg/mL streptomycin solution (ATCC^®^ 30–2300^™^) was used. Cells used in this study were between passages 4 and 7. The channels coated with 350 μg/mL collagen were injected with the cells for all the experiments. The cells were allowed to grow completely inside the channels for two days. Channels were kept in a Petri dish with medium for the exchange of nutrients. The growth and morphology of the cells were continuously monitored using an inverted microscope to ensure appropriate cellular morphology.

### 2.2. Flow System

A peristaltic pump (3384, Fisherbrand) was used to mimic the flow in the established blood vessel model. Phenol red was used as a substitute for blood for flow experiment due to the deposition of red blood cells when using blood. The channel was connected to tubes on both sides through smoothed needles inserted as connectors. The ends were then sealed with paraffin to avoid any contamination to the cells during experiments. The channel quality along with the flow system was examined by studying the number of cells detached from the channels at varying speeds of phenol red inside the blood vessel model: 2.5, 5, 10, and 20 mm/s with five samples for each speed. The flow experiment for each channel was also performed for 10 min only, as PUT treatment time was planned for the same time. The medium was collected after the experiment and the detached cells were counted using a hemocytometer.

### 2.3. Photoacoustic Signal of Medium Containing Phenol Red

Previous studies have demonstrated that the enhanced cavitation activity during PUT is based on the photoacoustic effect [3,4,69]. Therefore, it is imperative for the current in vitro model to produce similar photoacoustic signals as real blood vessels. In the current in vitro model, photoacoustic signals are produced due to the optical absorption of phenol red. To find out the optimal concentration of phenol red as a substitute in place of blood, the photoacoustic signal of 20% (*υ*/*υ*) and 25% (*υ*/*υ*) phenol red in the medium was compared to that of rhesus monkey whole blood containing sodium citrate as an anticoagulant (IGMNRSWBNAC10ML, Innovative Research, Novi, MI, USA). The channel along with the flow system was set up, and the photoacoustic signal was acquired using an oscilloscope when the solution was either 20% (*υ*/*υ*) or 25% (*υ*/*υ*) phenol red in medium or blood.

### 2.4. Ultrasound and Laser System Setup for Assessing NO and PGI_2_ Release

**Figure 1.**
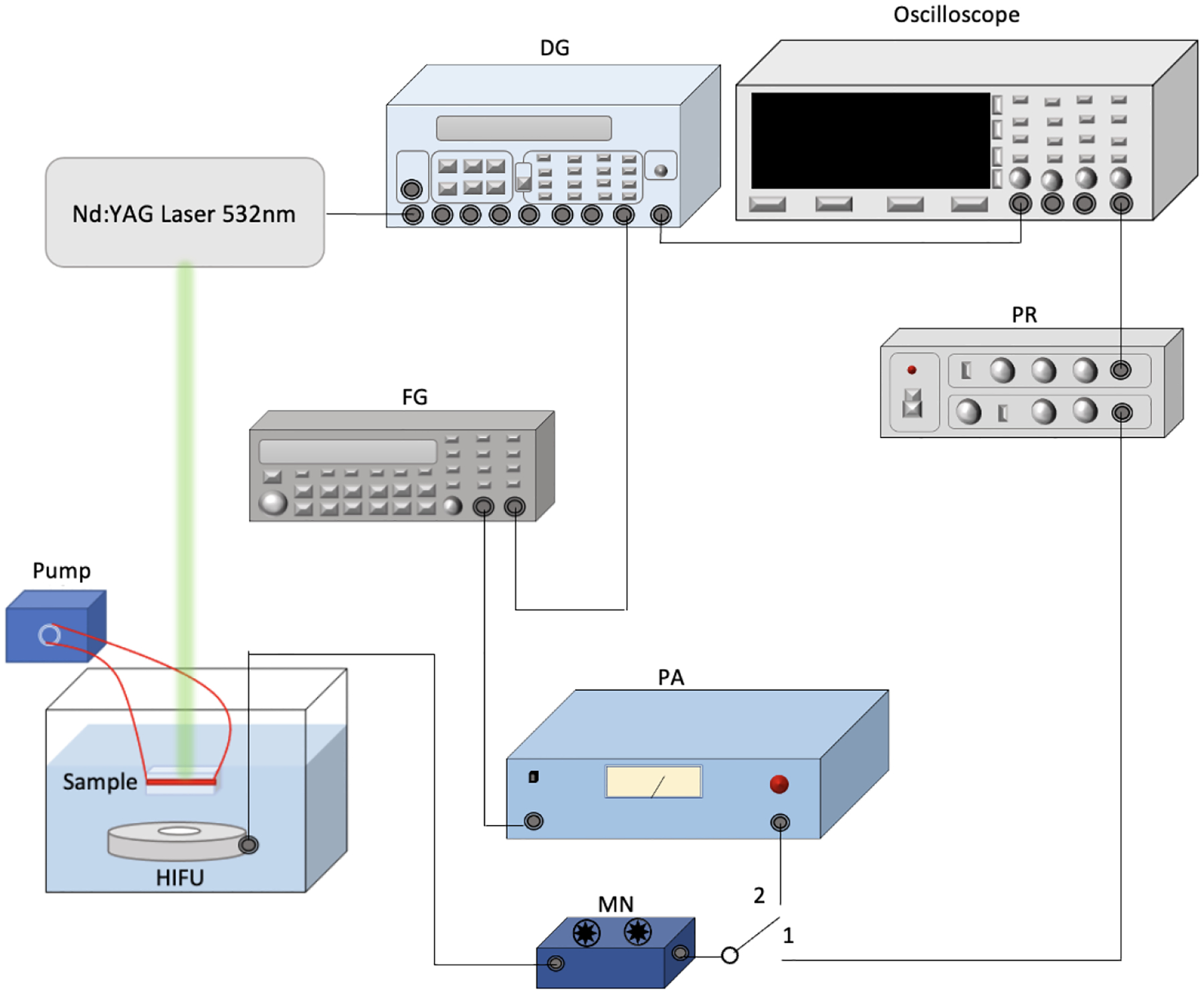
Schematic of the experimental setup for treatment in vitro. PA: power amplifier. DG: delay generator. PR: pulse-receiver. MN: matching network for HIFU transducer. FG: function generator. HIFU: high-intensity focused ultrasound. Position 1 is for detecting photoacoustic signals during the alignment process and Position 2 is for treatment.

The schematic of the experimental set up with ultrasound/laser and flow system is shown in Figure 1. A 532 nm laser system (Surelite SLI-30, Continuum, Santa Clara, CA, USA), with a pulse repetition rate of 30 Hz and a pulse duration of 5 ns, was used to produce photoacoustic signals and align the focal region of a 500 kHz high-intensity focused ultrasound (HIFU) transducer (H107, Sonic Concepts, Bothell, WA, USA) on the channel before each treatment. A pulse delay generator (Model DG355, Stanford Research Systems, Sunnyvale, CA, USA) was used to trigger the laser system, ultrasound system, and oscilloscope with a repetition rate of 30 Hz. During the alignment process (switch 1 position), the laser beam was used to illuminate the channel, and the produced photoacoustic signal was detected by using the HIFU transducer. The signal was acquired by a digital oscilloscope (DPO 3034, Tektronix Inc., Beaverton, OR, USA). The HIFU transducer was scanned across the channel until the maximum photoacoustic signal was detected, indicating the focal region of the HIFU transducer was on the channel and overlaid the laser beam.

During treatment (switch 2 position), a function generator (33250A, Agilent Technologies, Santa Clara, CA, USA) supplied ultrasound signals. Its output was first amplified by 50 dB using a power amplifier (350L RF Power Amplifier, ENI Technology Inc., Rochester, NY, USA). It was then connected to the HIFU transducer through an impedance matching circuit (Impedance Matching Network H107, Sonic Concepts, Bothell, WA, USA). The focal spot of the HIFU transducer, whose focal length and focal width are 21.02 and 3.02 mm, respectively, was focused at the center of the designated channel. The radius of curvature of the HIFU transducer is 63.2 mm. Ultrasound bursts with 10% duty cycle were used to minimize the generation of heat. Each burst contained 1650 cycles and wae repeated at a 30 Hz pulse repetition rate.

For PUT treatment, the laser intensities were carefully controlled to the desired laser fluence on the sample surface, and the same ultrasound parameters used in ultrasound-only treatments were used. In PUT, laser treatment and ultrasound were applied synchronously [6]. The laser spot diameter used for the treatment was 6 mm. An optical power meter was used to check the laser power before treatment for each sample.

For NO measurement, the treatment pressures were 0.1, 0.5, 1, 1.5, and 2 MPa in ultrasound-only treatment group, and laser fluences were 5, 10, 15, and 20 mJ/cm^2^ in laser-only treatment group with six samples (*n* = 6) in each group. In the first PUT group (*n* = 9), the 0.1, 0.5, and 1 MPa ultrasound pressures and laser intensity of 10 mJ/cm^2^ were applied synchronously. In the second PUT group (*n* = 6), the 5, 10, 15, and 20 mJ/cm^2^ laser fluences and ultrasound intensity of 0.5 MPa were applied synchronously.

For PGI_2_ measurement, the treatment pressures were 0.5, 1, 1.5, and 2 MPa in ultrasound-only treatment group, and laser fluences were 5, 10, 15, and 20 mJ/cm^2^ in laser-only treatment group. The same laser fluence range and ultrasound pressure of 0.5 MPa were applied synchronously in PUT group with six samples (*n* = 6) in each group. As the change in NO began to decrease from 1.5 MPa for ultrasound only group, 0.1 MPa for PCI_2_ measurements was not considered. Moreover, when changing ultrasound for constant laser, there was no significant change in any group except 0.1 MPa. Hence, the variation in ultrasound pressure for constant laser fluence was not carried out for PCI_2_ measurements.

For all treatment groups except the control, each channel was divided into four spots of identical length covering the entire channel and each spot was treated for 2.5 min. After the treatment, the medium was immediately collected and stored at −80 °C until the assay was performed. As the volatile NO gets converted into nitrite and nitrate, the total NO (KGE001, R&D Systems, Minneapolis, MN, USA) was measured through nitrate reduction assay with Griess reagents I and II. The PGI_2_ was measured through urinary PGI_2_ ELISA kit (NBP2–62171, Novus Biologicals, Littleton, CO, USA). Both assays were measured using a Cytation 5 (BioTek Instruments Inc., Winooski, VT, USA).

### 2.5. Nitrate Reduction Assay

Total NO assay kit was used for nitrate reduction assay (R&D Systems, Minneapolis, MN, USA). All standard dilutions, reagents and samples were prepared as per the manufacturer’s instructions. Briefly, excess microplate strips were first removed from the plate frame. Then, 50 μL of reaction diluent was added to the blank wells. A total of 50 μL of standard dilutions or samples was then added to these wells. After that, 25 μL of reduced nicotinamide adenine dinucleotide (NADH) was added to all wells, followed by 25 μL of nitrate reductase. The plate was then sealed with a plate sealer and incubated at 37 °C for 30 min. In total, 50 μL of Griess reagent I was added. Finally, 50 μL of Griess reagent II was added, and the plate was incubated at room temperature for 10 min. The absorbance was read at 540 nm with a correction wavelength at 690 nm.

### 2.6. PGI_2_ Assay

Non-species specific PGI_2_ ELISA kit was used for PGI_2_ assay (Novus^™^ Biologicals, Littleton, CO, USA). All standard dilutions, reagents and samples were prepared as per the manufacturer’s instructions. Briefly, excess microplate strips were first removed from the plate frame. Then, 100 μL of standard diluent was added to the blank and non-specific binding wells. In total, 100 μL of standard dilutions or samples was then added to the remaining wells. Then, 50 μL of assay buffer was added to non-specific binding wells. After that, 50 μL of blue conjugate was added to standard, samples, and non-specific binding wells. A total of 50 μL of yellow conjugate was added to standard and samples wells. The plate was then sealed with a plate sealer and incubated at room temperature on a plate shaker for 2 h at ~500 rpm. The contents of the well were emptied and washed with wash solution. The wells were emptied and tapped on a lint-free paper towel after the final wash. Then, 5 μL of blue conjugate diluted 1:10 in assay buffer was added to the total activity well. A total of 200 μL of p-NPP substrate was added to all the wells and incubated at room temperature for 45 min without shaking. Finally, 50 μL of stop solution was added, and the plate was read immediately. The absorbance was read at 405 nm with a correction wavelength at 570 nm.

### 2.7. Caυitation Detection

A passive cavitation detector (PCD) was used to detect the signals generated from the focal zone of the 0.5 MHz HIFU transducer. A 10 MHz center frequency focused ultrasound transducer (V315, Olympus NDT, Waltham, MA, USA) was used as a PCD to have more sensitivity at the high-frequency broadband noise. The PCD was aligned confocal to the HIFU transducer such that both of their focal zones were overlying. The PCD was connected to a pulse receiver (DPR300, JSR Ultrasonics, Pittsford, NY, USA) which functioned as a high pass filter of 1 MHz and was used to reduce the interference signal from the 0.5 MHz HIFU transducer. The pulse receiver also amplified the detected signal by 30 dB before being acquired by the personal computer through a DAQ card. A cavitation event was identified when the amplitude of the detected signal was at least twice that of the noise baseline.

### 2.8. Apoptosis Detection

Apoptosis was studied using eBioscience^™^ Annexin V Apoptosis Detection Kit FITC (88–8005, Invitrogen). Then, 10× binding buffer was diluted to 1× buffer using distilled water. The cells were washed once with PBS and then washed with the binding buffer. The cells were incubated with 5 μL of fluorochrome-conjugated Annexin V in 100 μL of 1× binding buffer for 15 min at room temperature. The cells were then washed with 1× binding buffer. The cells were then incubated in the dark at 2–8 degrees Celsius with 5 μL of propidium iodide staining solution in 200 μL of 1× binding buffer. The cells were then observed under fluorescence microscope within 4 h.

### 2.9. Statistical Analysis

SPSS software version 27.0 (SPSS Inc., Chicago, IL, USA) was used to perform statistical analysis. Student’s *t*-test was used to compare significant difference in means of independent data. Variance is expressed as mean ± standard error of the mean. A constant 2-tailed *p*-value of less than 0.05 was used to denote statistical significance. In the results section, “*” indicates statistical significance between groups with *p* < 0.05. “**” indicates statistical significance between treatment groups with *p* < 0.01. “***” indicates statistical significance between groups with *p* < 0.001. “†” indicates statistical significance between control and treatment group *p* < 0.05. “††” indicates statistical significance between control and treatment group *p* < 0.01. “†††” indicates statistical significance between control and treatment group *p* < 0.001.

## 3. Results

### 3.1. Flow System

The channel quality in maintaining the integrity of the cells during the treatment with ultrasound was evaluated in cell detachment experiments. The cell detachment was found to increase with the increased flow speed of the solution inside the channel, as shown in Figure S2. The mean number of cells detached from the channel with different flow speeds varied from ~100 cells for the lower speed of 2.5 mm/s, to ~1100 cells for the highest speed of 20 mm/s. The average number of cells in each channel were ~200,000, resulting in the percentages of detached cells as 0.04%, 0.22%, 0.40% and 0.53% for 2.5, 5, 10, and 20 mm/s flow speed, respectively.

### 3.2. Photoacoustic Signal of Medium Containing Phenol Red

**Figure 2.**
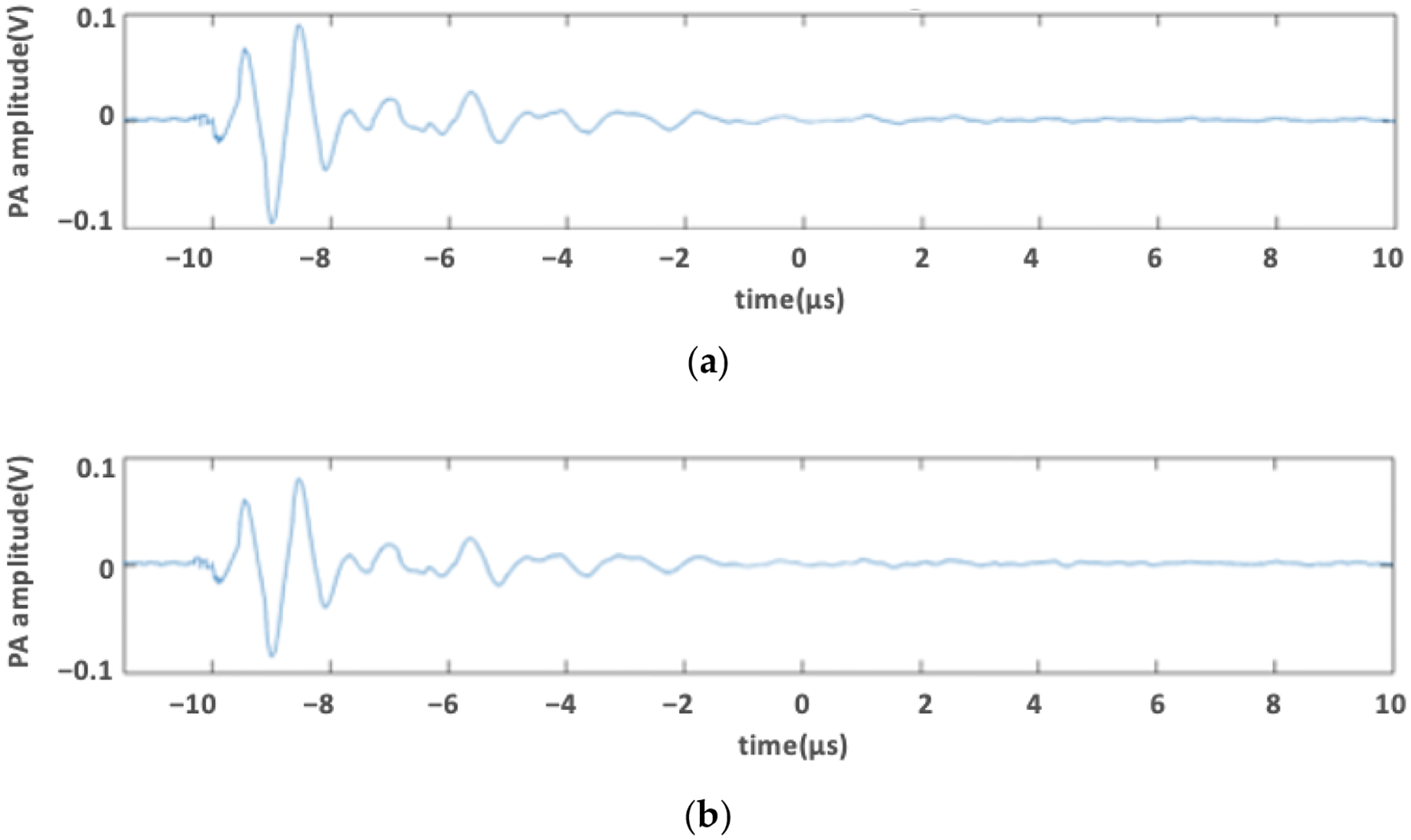
Photoacoustic signal of (**a**) blood and (**b**) 25% (*υ*/*υ*) phenol red in medium acquired for laser fluence of 0.77 mJ/cm^2^ at 532 nm laser wavelength.

To prove the efficiency of the in vitro model in mimicking the blood vessel for its application in PUT, we acquired photoacoustic signals of two different concentrations of phenol red in complete medium: 20% (*υ*/*υ*) and 25% (*υ*/*υ*). The photoacoustic signals were compared to that of the blood, as shown in Figure 2. The photoacoustic signal amplitude acquired for 25% (*υ*/*υ*) phenol red in the complete medium was closer to that observed from the blood. However, the photoaooustic signal produced by the 20% (*υ*/*υ*) phenol red in the complete medium was too weak compared to that observed from the blood. Moreover, increasing the concentration above 25% (*υ*/*υ*) did not change the amplitude of the photoacoustic signal. Hence, we used 25% (*υ*/*υ*) phenol red in the complete medium for experimental validation of the in vitro model.

### 3.3. Effect of Ultrasound-Only Treatment on NO Level

**Figure 3.**
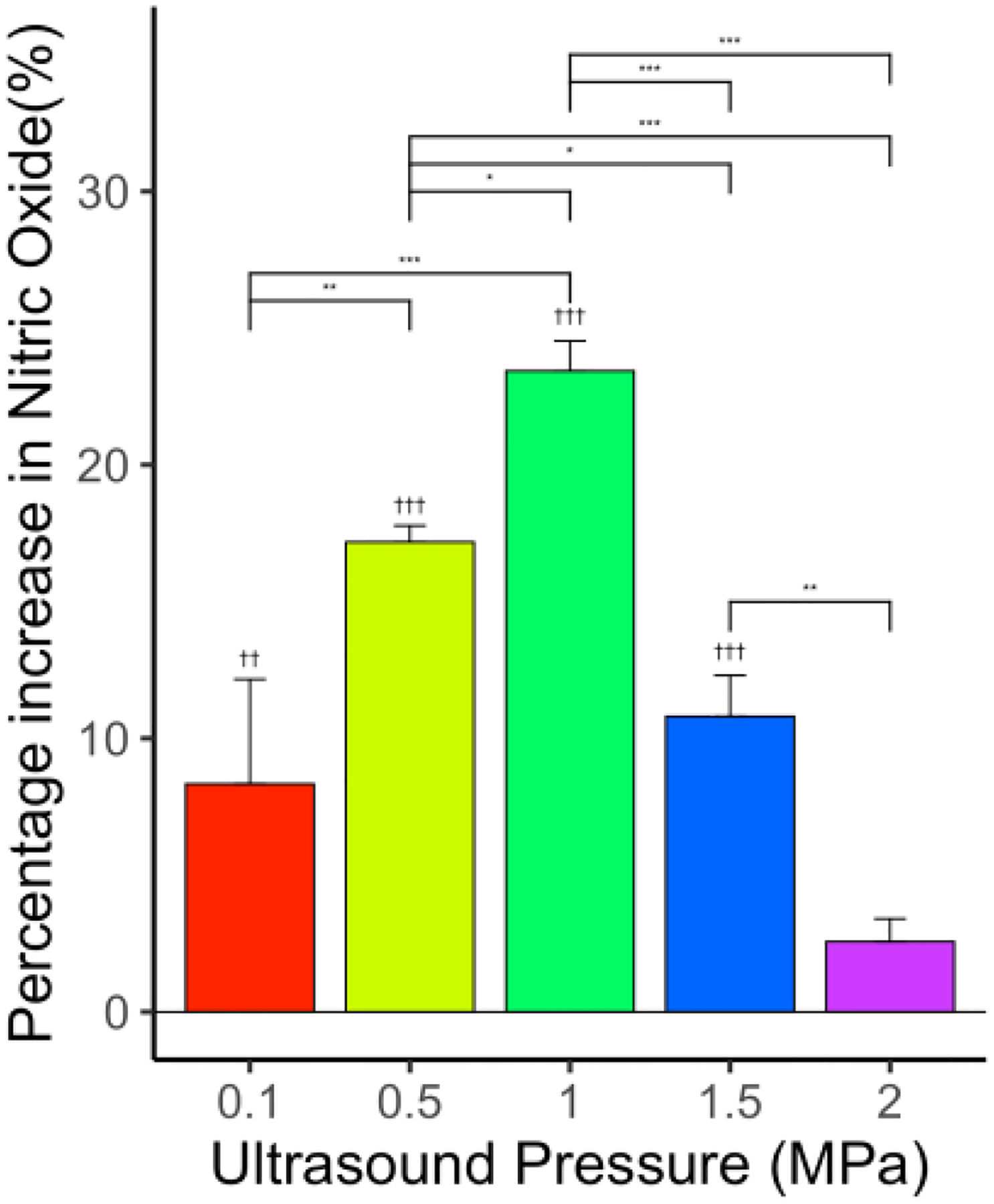
Effect of US on NO levels for various levels of ultrasound peak negative pressures—0.1, 0.5, 1, 1.5, and 2 MPa—showing significant increase in NO levels compared to control. *N* = 6 for each group. “*” indicates statistical significance between groups with *p* < 0.05. “**” indicates statistical significance between treatment groups with *p* < 0.01. “***” indicates statistical significance between groups with *p* < 0.001. “††” indicates statistical significance between control and treatment group *p* < 0.01. “†††” indicates statistical significance between control and treatment group *p* < 0.001.

**Figure 4.**
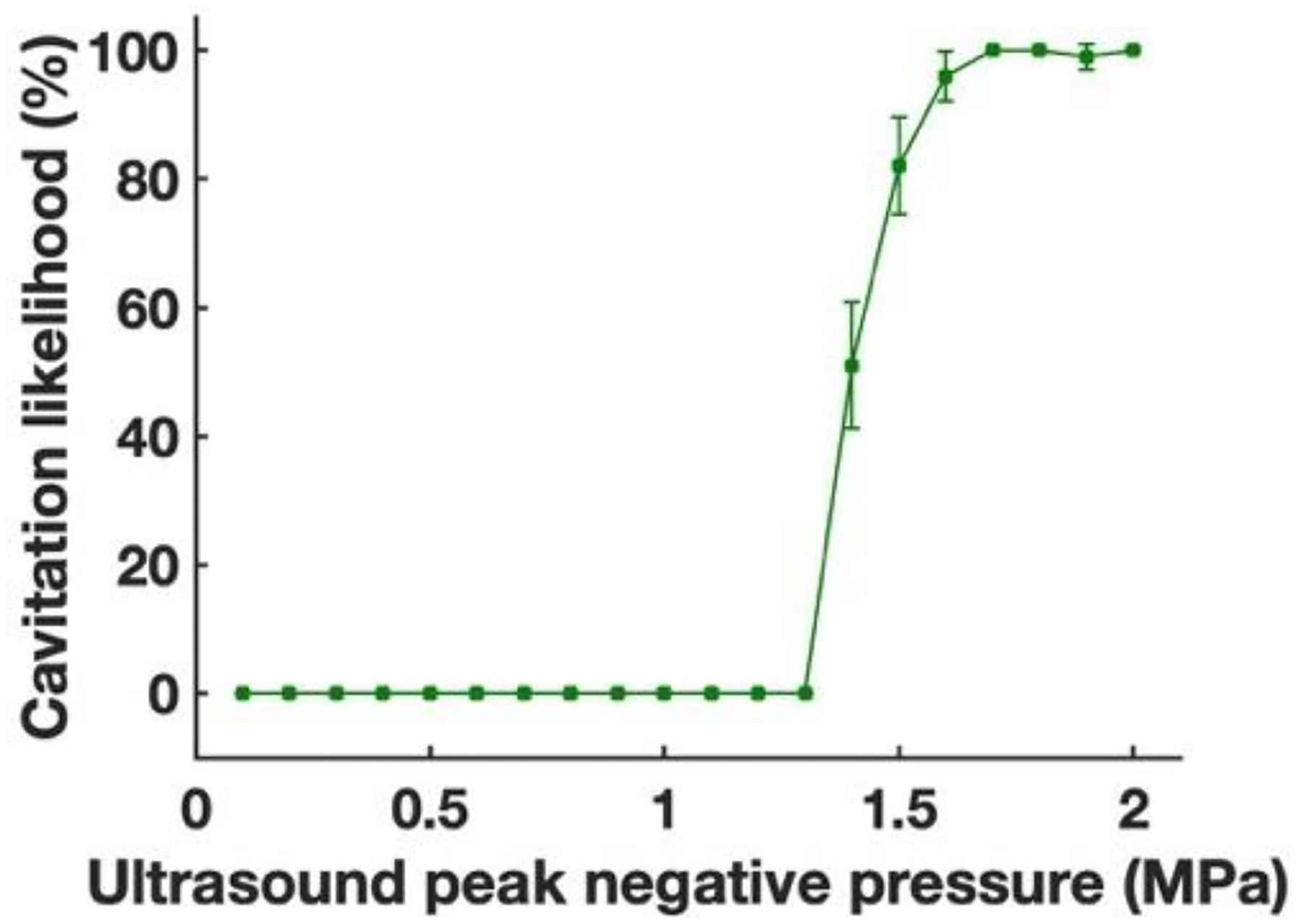
Cavitation likelihood detected using passive cavitation detector.

Since, in the initial experiments, using blood caused adhesion of red blood cells to the channel wall coated with endothelial cells, we replaced blood with the complete medium containing phenol red [47]. The percentage increase in the NO level in comparison with the control group was 8.3% (*p* < 0.01, *n* = 6), 17.2% (*p* < 0.001, *n* = 6), 23.4% (*p* < 0.001, *n* = 6), 10.8% (*p* < 0.001, *n* = 6), and 2.5% for 0.1, 0.5, 1, 1.5, and 2 MPa, respectively, as shown in Figure 3. The NO level increased by 181.9% when ultrasound peak negative pressure levels increased from 0.1 to 1 MPa, and then the NO level was reduced by 89.3% when ultrasound peak negative pressure increased from 1 to 2 MPa. Figure 4 shows the PCD measured cavitation likelihood as a function of ultrasound peak negative pressure. A strong cavitation signal was detected when the ultrasound peak negative pressure was greater than 1.3 MPa, which indicates the threshold for inertial cavitation (Figure S3). This could explain the reduced NO level at high ultrasound peak negative pressure. However, the overall NO level was still higher than the control group within the selected parameter range, indicating that not all endothelial cells are dysfunctional at the selected ultrasound pressure. Impairment of endothelial-dependent relaxations results in reduced endothelium-derived NO synthesis, which is a characteristic of endothelial cell dysfunction [70].

### 3.4. Effect of Laser-Only Treatment on NO Level

**Figure 5.**
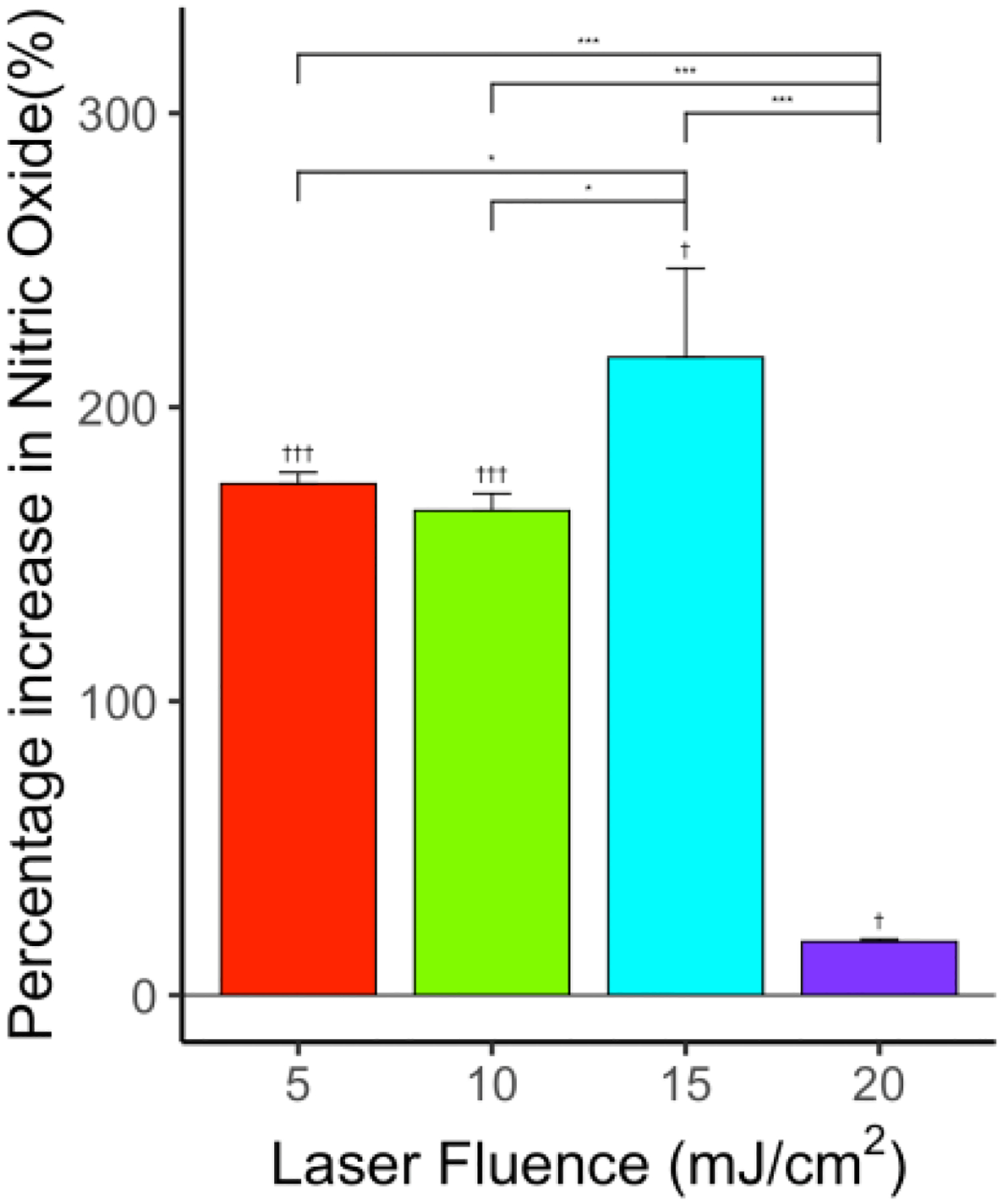
Effect of laser on NO levels for various levels of laser fluence—5, 10, 15 and 20 mJ/cm^2^—showing significant increase in NO levels compared to control. *n* = 6 for each group. “*” indicates statistical significance between groups with *p* < 0.05. “***” indicates statistical significance between groups with *p* < 0.001. “†” indicates statistical significance between control and treatment group *p* < 0.05. “†††” indicates statistical significance between control and treatment group *p* < 0.001.

The percentage increase in NO level in comparison with the control group was 173.9% (*p* < 0.001, *n* = 6), 164.8% (*p* < 0.001, *n* = 6), 216.9% (*p* < 0.05, *n* = 6), and 18.2% (*p* < 0.05, *n* = 6) for 5, 10, 15, and 20 mJ/cm^2^ laser fluence, respectively, as shown in Figure 5. The increase in NO level is consistent with previous studies indicating that a low-intensity laser can induce increased NO release from endothelial cells [71,72]. Similar to ultrasound-only treatment, the enhancement in NO level was significantly decreased at a high laser fluence of 20 mJ/cm^2^ (*p* < 0.001, *n* = 6) in comparison with all other laser-only treatment groups, indicating that endothelial cell dysfunction might have occurred at the high energy treatment level.

### 3.5. Effect of Photo-Mediated Ultrasound Therapy on NO Level

#### 3.5.1. Constant Ultrasound with Varying Laser Fluence

**Figure 6.**
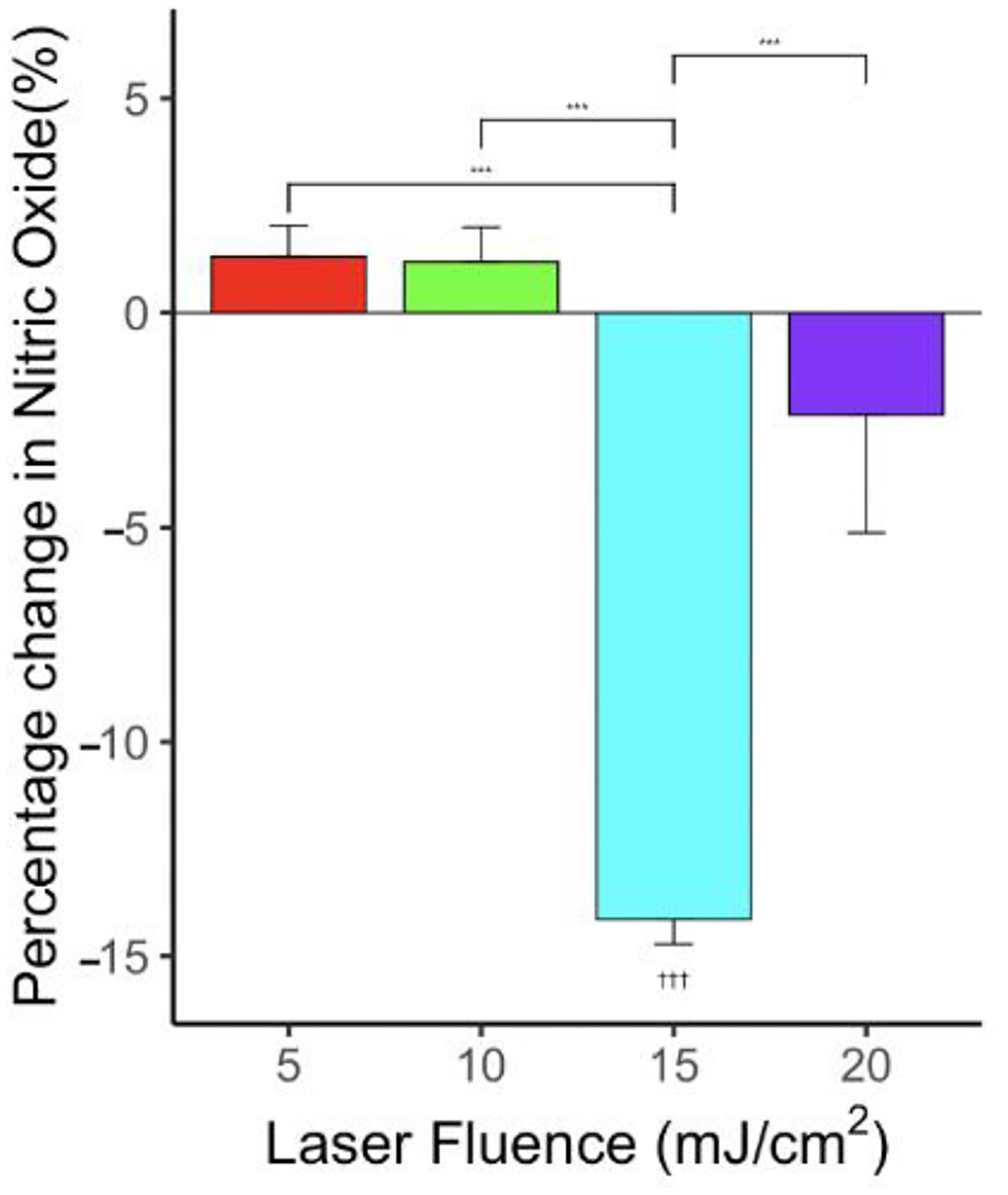
Effect of PUT on NO levels for various laser fluences—5, 10, 15, and 20 mJ/cm^2^—applied synchronously with ultrasound = 0.5 MPa showing significant change in NO levels compared to control. *n* = 6 for each group. “***” indicates statistical significance between groups with *p* < 0.001. “†††” indicates statistical significance between control and treatment group *p* < 0.001.

While ultrasound-only with 0.5 MPa showed an enhanced NO level in Figure 3, there was no significant change in NO level compared to the control when ultrasound peak negative pressure 0.5 MPa was applied synchronously with 5, 10, and 20 mJ/cm^2^ laser fluence, as shown in Figure 6. Furthermore, there is a statistically significant decrease in NO level measured at 15 mJ/cm^2^ laser fluence when synchronized with 0.5 MPa ultrasound (*p* < 0.001, *n* = 6). The fast Fourier transform (FFT) of the PCD signal indicated subharmonic (refer Supplementary Figure S4) signals, which is the hallmark of oscillating cavitation bubbles, at 10 mJ/cm^2^ when synchronized with 0.5 MPa ultrasound. This result indicated that the strong cavitation activities during PUT might have caused endothelial cell dysfunction, and, as a result, the NO level was reduced.

#### 3.5.2. Constant Laser Fluence with Varying Ultrasound Pressure

**Figure 7.**
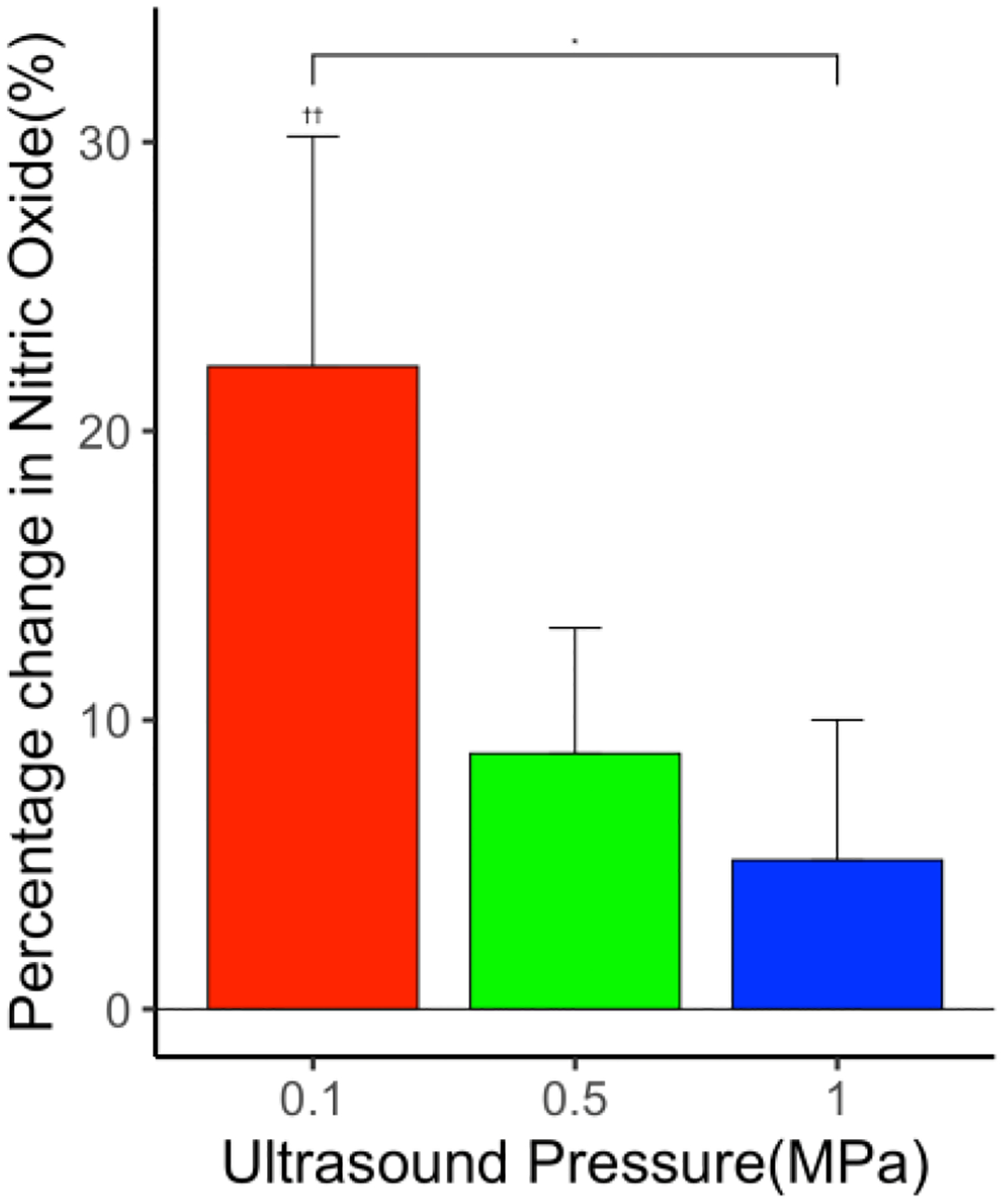
Effect of PUT on NO levels for various levels of ultrasound peak negative pressuros—0.1, 0.5, and 1 MPa—applied with laser = 10 mJ/cm^2^ showing increase in NO levels compared to control. *n* = 9 for each group. “*” indicates statistical significance between groups with *p* < 0.05. “††” indicates statistical significance between control and treatment group *p* < 0.01.

When the laser fluence was fixed at 10 mJ/cm^2^ and the applied ultrasound peak negative pressure was increased, the overall NO level was increased. But the level of increase was significantly reduced with ultrasound peak negative pressure increasing from 0.1 to 1 MPa (*p* < 0.05, *n* = 9) (Figure 7). Again, enhanced cavitation activities-induced endothelial cell dysfunction or cell death could be the reason for the NO level reduction (Figures S5–S7).

### 3.6. Effect of Ultrasound-Only Treatment on PGI_2_ Level

**Figure 8.**
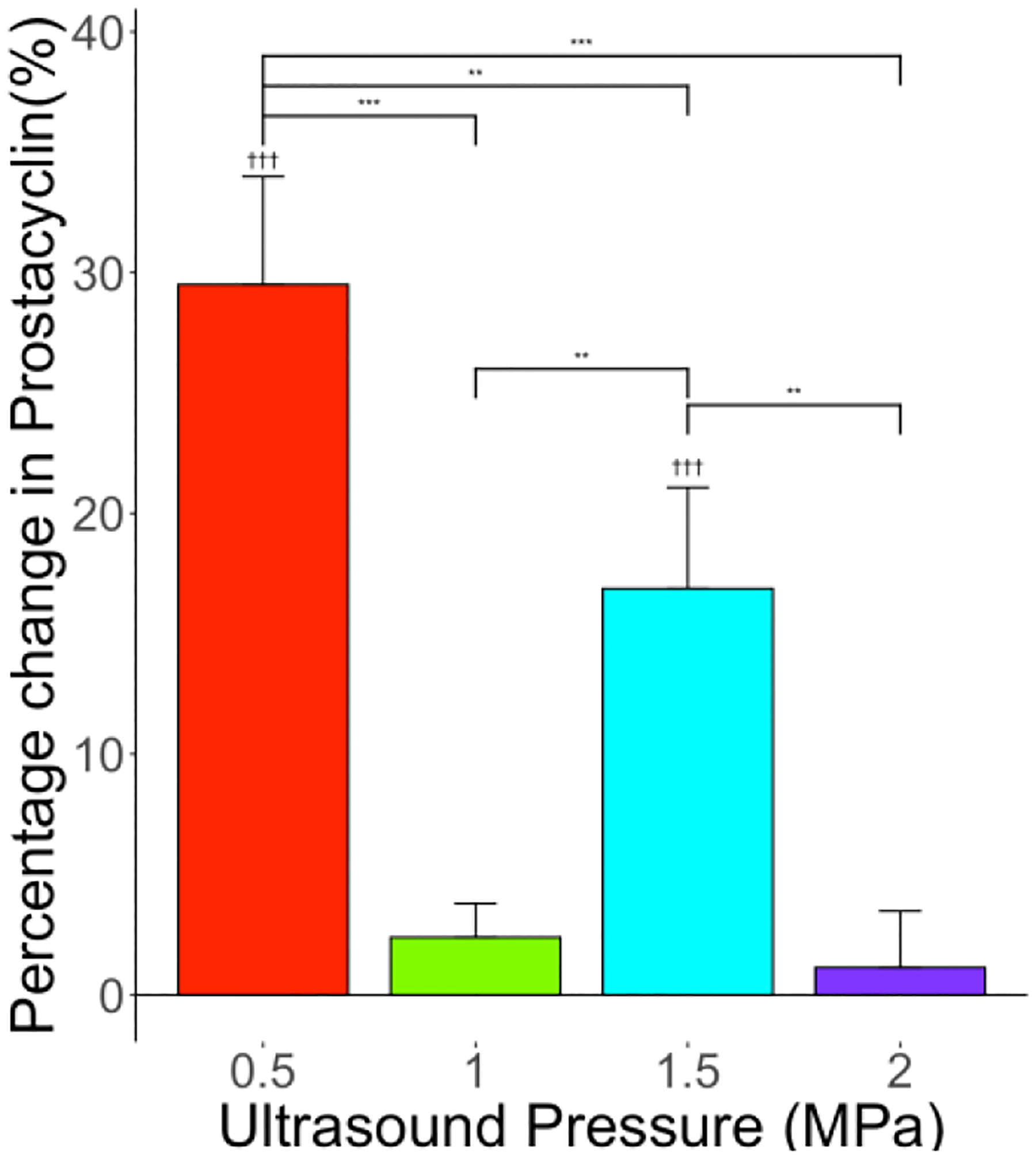
Effect of US on PGI_2_ levels for various levels of ultrasound peak negative pressures—0.5, 1, 1.5, and 2 MPa—showing significant change in PGI_2_ levels compared to control. *n* = 6 for each group. “**” indicates statistical significance between treatment groups with *p* < 0.01. “***” indicates statistical significance between groups with *p* < 0.001. “†††” indicates statistical significance between control and treatment group *p* < 0.001.

The applied ultrasound induced an increase in PGI_2_ level. The percentage increase in PGI_2_ level in comparison with the control group was 29.5% (*p* < 0.001, *n* = 6), 2.4%, 16.9% (*p* < 0.001, *n* = 6), and 1.14% for the ultrasound-only treatment groups 0.5, 1, 1.5, and 2 MPa, respectively, as shown in Figure 8. While the overall level of PGI_2_ increased in comparison with the control, there is a general trend of reduction in the level of increase with increased ultrasound pressure. Again, the reduction in the level of increase in PGI_2_ could be an effect of cavitation activities in high ultrasound intensities. At the low-intensity ultrasound level, the release of PGI_2_ increases.

### 3.7. Effect of Laser-Only Treatment on PGI_2_ Level

**Figure 9.**
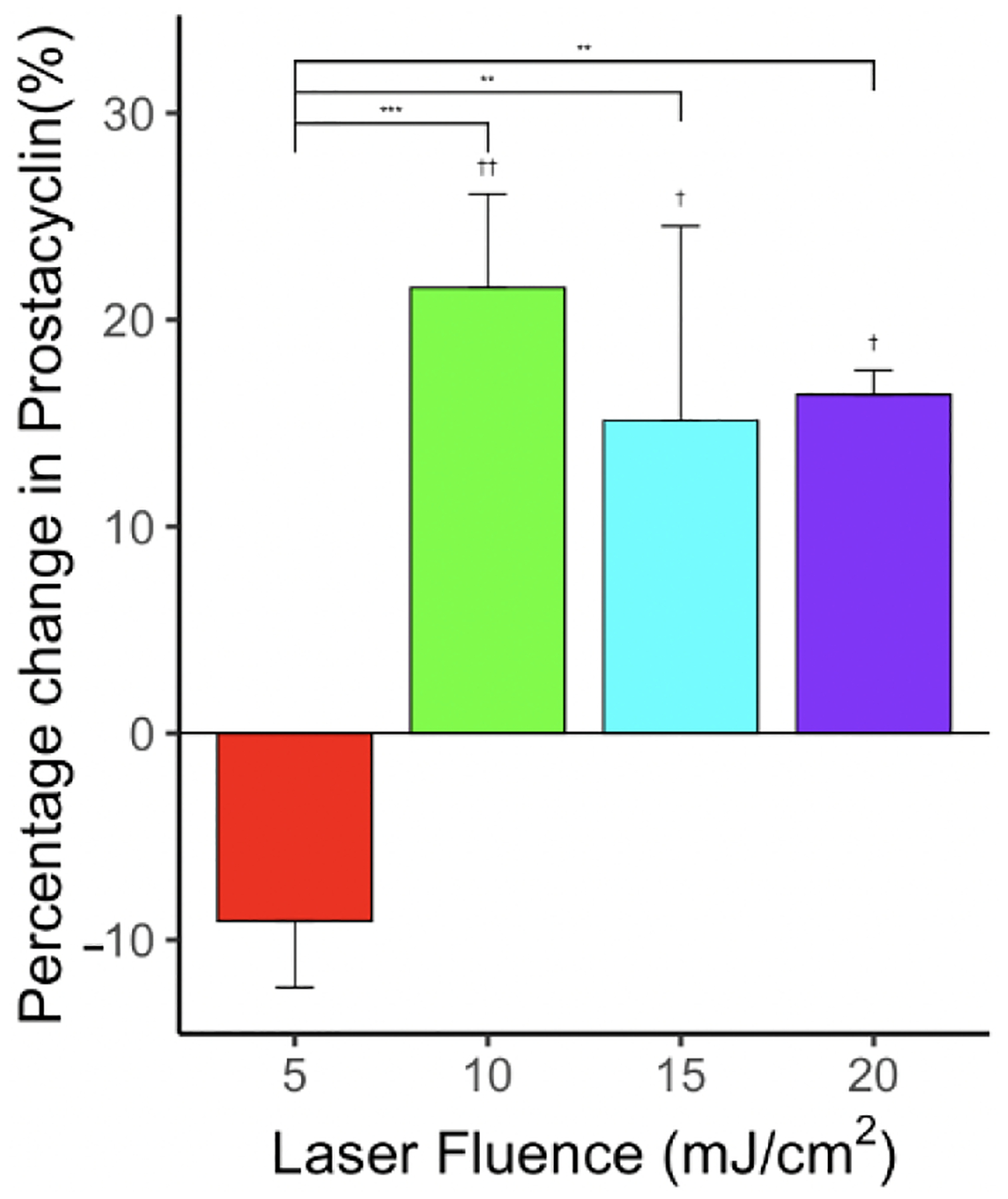
Effect of laser on PGI_2_ levels for various laser fluences—5, 10, 15, and 20 mJ/cm^2^—showing statistically significant changes in PGI_2_ levels compared to control. *n* = 6 for each group. “**” indicates statistical significance between treatment groups with *p* < 0.01. “***” indicates statistical significance between groups with *p* < 0.001. “†” indicates statistical significance between control and treatment group p < 0.05. “††” indicates statistical significance between control and treatment group *p* < 0.01.

The overall level of PGI_2_ increased when only laser energy was applied. At a low fluence level (5 mJ/cm^2^), there was a slight decrease (not statistically significant) in PGI_2_ level. At high fluence levels, the percentage increase in PGI_2_ level in comparison with the control group was 21.6% (*p* < 0.01, *n* = 6), 15.1% (*p* < 0.05, *n* = 6), and 16.4% (*p* < 0.05, *n* = 6) for 10, 15, and 20 mJ/cm^2^, respectively, as shown in Figure 9. The percentage increase in PGI_2_ level was statistically significant between the treatment group 5 mJ/cm^2^ and all other laser-only treatment groups 10 mJ/cm^2^ (*p* < 0.001, *n* = 6), 15 mJ/cm^2^ (*p* < 0.01, *n* = 6) and 20 mJ/cm^2^ (*p* < 0.01, *n* = 6), respectively. Low power laser density was shown to increase the level of PGI_2_ [73].

### 3.8. Effect of Photo-Mediated Ultrasound Therapy on PGI_2_ Level

**Figure 10.**
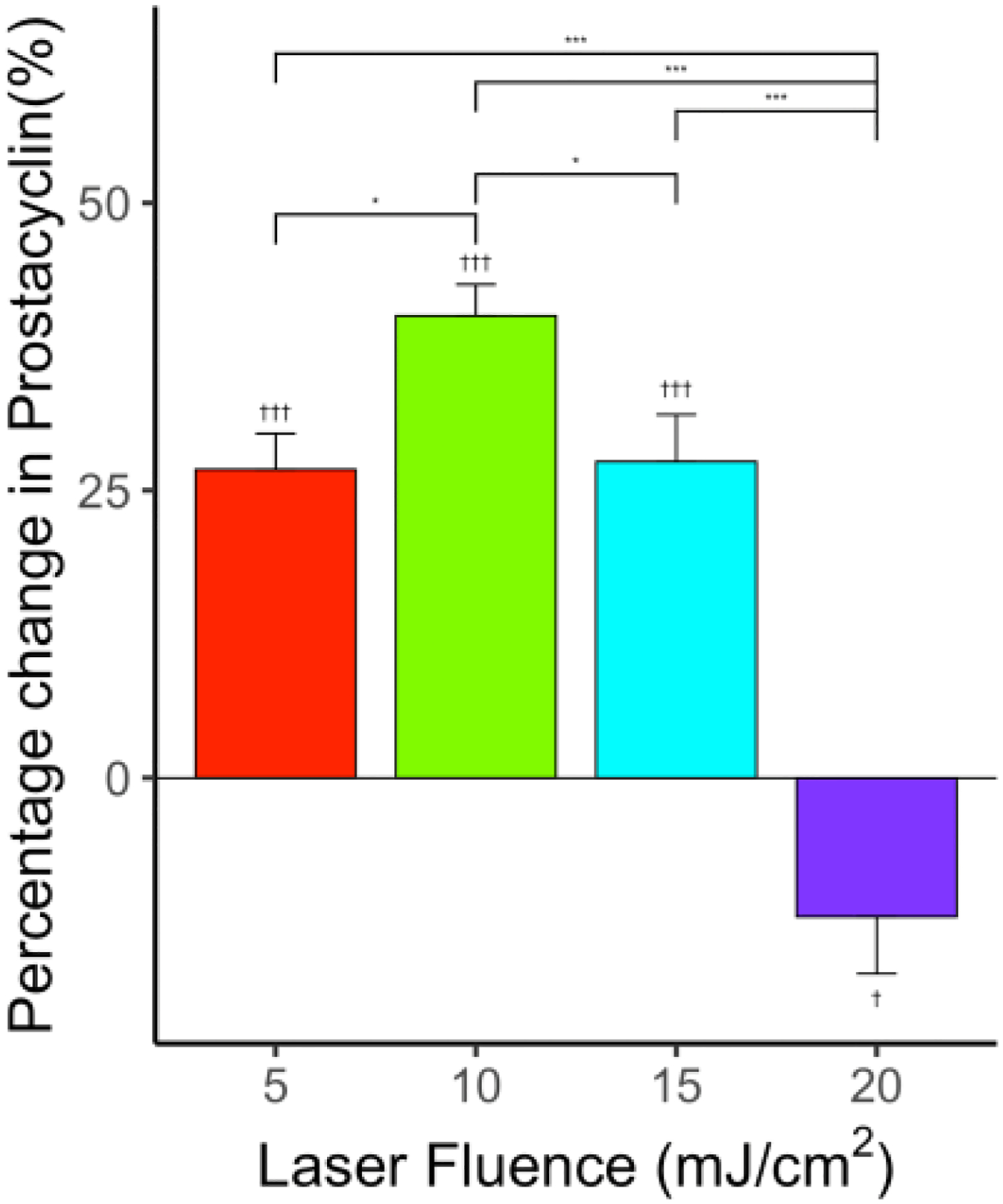
Effect of PUT on NO levels for various laser fluences—5, 10, 15, and 20 mJ/cm^2^—applied synchronously with ultrasound = 0.5 MPa showing a significant change in PGI_2_ levels compared to control. *n* = 6 for each group. “*” indicates statistical significance between groups with p < 0.05. “***” indicates statistical significance between groups with *p* < 0.001. “†” indicates statistical significance between control and *p* < 0.05. “†††” indicates statistical significance between control and treatment group p < 0.001.

With PUT, the level of PGI_2_ showed an increase at the low energy level and a clear decrease at the high energy level (both are statistically significant). The percentage increase in PGI_2_ level in comparison with the control group was 26.8% (*p* < 0.001, *n* = 6), 40.2% (*p* < 0.001, *n* = 6), and 27.5% (*p* < 0.001, *n* = 6) for laser fluences 5, 10, and 15 mJ/cm^2^ when applied synchronously with 0.5 MPa ultrasound peak negative pressure, respectively, as shown in Figure 10. At the highest laser fluence, the level of PGI_2_ decreased in comparison with the control group (−12.1%, *p* < 0.001, *n* = 6) for laser fluences 20 mJ/cm^2^ when applied synchronously with 0.5 MPa ultrasound peak negative pressure. At this energy level, the PCD result showed a clear subharmonic signal, indicating the presence of cavitation bubbles in the vessel.

**Figure 11.**
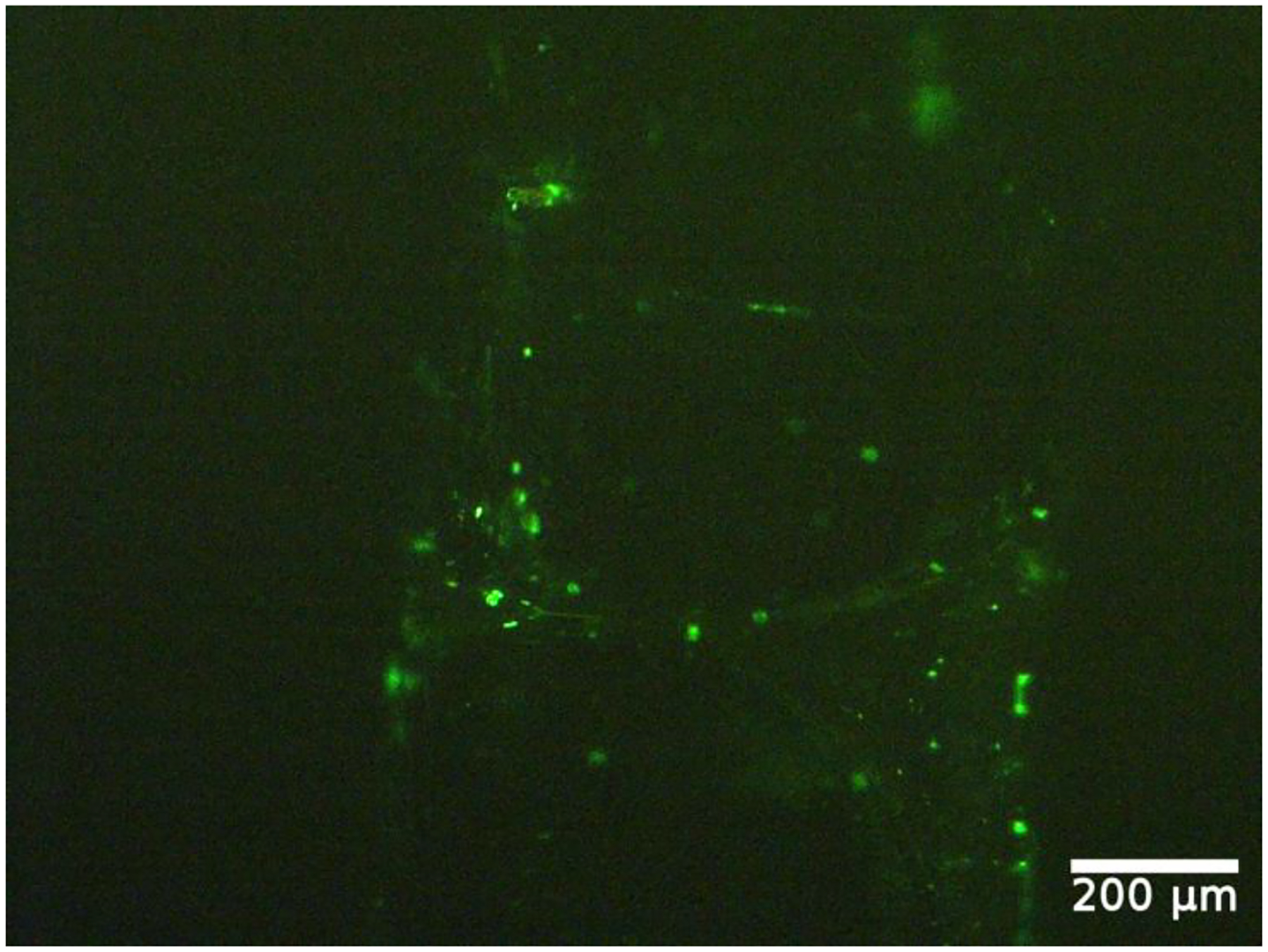
Apoptosis of endothelial cells after being treated with 0.5 MPa ultrasound peak negative pressure and 15 mJ/cm^2^ laser fluence. Apoptotic cells show green fluorescence, and live cells show little or no fluorescence.

The apoptosis of cells when treated with 0.5 MPa ultrasound peak negative pressure and 15 mJ/cm^2^ laser fluence is shown in Figure 11. Green fluorescence indicates apoptosis in cells while live cells show little or no fluorescence. This result confirms that the dysfunction may have occurred in some endothelial cells. Subsequently, the levels of NO and PCI_2_ are significantly affected by the endothelial cell dysfunction, which can explain the observed reduction in the levels of NO and PCI_2_.

## 4. Discussion

Chorioretinal endothelial cells from rhesus monkeys were used in this study. The rhesus monkey eye was found to resemble the human eye in various aspects. Mainly, the retina possesses a macula common only in monkeys, humans, and apes. The macula in rhesus monkeys and humans contains macular pigment, a foveal pit consisting of the maximum concentration of cones, a capillary-free region, and the dislocation of inner retinal layers and vessels. Further, rhesus monkeys and humans were also found to share common genes susceptible to age-related macular degeneration [74].

The Griess method is specific for measuring nitrite [75]. NO is a gaseous free radical with a half-life of a few seconds or less. So, NO is indirectly measured in the cell culture supernatant through the levels of the more stable NO metabolites, nitrite (NO_2_) and nitrate (NO−) [76–79]. The mean minimum detectable dose was 0.25 μmol/L for the kit used. In this study, the total NO was measured using the Griess method by converting nitrate into nitrite using nitrate reductase. The nitrite was then detected as an azo dye through calorimetric detection [80,81]. The PGI_2_ kit used was non-species specific. The polyclonal antibody in the kit binds competitively in equal affinities with 6-keto-Prostaglandin F1*α* (6k-PGF1*α*) and 2,3-dinor-6-keto-Prostaglandin F1*α* (2,3d-6k-PGF1*α*) [82].

In the current study, we used phenol red solution as a blood substitute to provide optical absorption for PUT. The phenol red solution was previously used in studying the role of ultrasound in the biochemical production of endothelial cells [47]. The use of phenol red can avoid the blood flowing inside the channels to cause the sticking of blood cells to the channel wall. The spatial-peak temporal-average ultrasound intensities used in this study were 0.033, 0.833, and 3.333 W/cm^2^, which are at the same order of magnitude as previous studies, and the increase in NO was obvious even at the lowest intensity ultrasound [71]. A previous study demonstrated that the NO level from endothelial cells will increase under low ultrasound pressures [72], which is consistent with our result from 0.1 to 1 MPa. The reduction in the NO level for ultrasound pressure from 1 to 2 MPa is likely due to the effect of strong cavitation activities, which can cause the death of endothelial cells [3], as demonstrated by the increase in apoptotic cells. Some inconsistencies in the measurement results, particularly at the high energy levels, could be an inherent feature of the cavitation-related effect. The cavitation threshold was at 1.3 MPa for ultrasound-only. Similarly, the cavitation was observed for the 15 mJ/cm^2^ and 0.5 MPa treatment group. Near or above the cavitation threshold, excessive cell death may occur locally. Because of the erratic behavior of cavitation, the results can vary in a large range.

Our measurement showed that: (1) at a low energy level, ultrasound-only and laser-only generally increased the release of NO and PGI_2_; and (2) ultrasound-only at a high energy level, or the combined laser and ultrasound (PUT), suppressed the release of NO and PGI_2_. This reduced release of NO and PGI_2_ could be correlated with cavitation activities in the vessel. Based on previous studies, the effect of PUT is primarily through the mechanical effect of the ultrasound wave, particularly cavitation. During PUT, the ultrasound pressure provided directly by the ultrasound transducer is superimposed by photoacoustic pressure, produced through the photoacoustic effect when the laser illuminated an optical absorptive sample. The superimposition of photoacoustic wave and the applied ultrasound wave can produce a stronger mechanical effect to activate endothelial cells. However, strong cavitation produced by PUT can damage endothelial cells, as demonstrated in the previous study [3], resulting in the dysfunction of endothelial cells and thus reduced levels of NO and PGI_2_ released. This can explain our observation in Figure 11 showing the apoptosis of cells.

Vallance et al. showed that on inhibiting the production of endothelial NO, a substantial fall in resting flow and an elevation in peripheral resistance was observed in the human forearm [83]. Reduced production of PGI_2_ was shown to result in the accumulation of plaques in blood vessels, which might result in the shutting down of the blood vessels [84,85]. As PUT was already shown to be capable of shutting the blood vessels, this study focuses on understanding the effects on the release of NO and PGI_2_ as they are vasoactive agents [4]. The changes in these levels were only expected to be associated with endothelial dysfunction.

PUT achieves therapeutic effect through cavitation produced in the blood vessel, so monitoring cavitation can provide more detailed information into the mechanism. However, the most frequently used passive cavitation detection technique is more sensitive in detecting inertial cavitation, which is more likely to damage endothelial cells. While we did observe inertial cavitation at a high energy level, we expect that the apoptosis of endothelial cells is more likely caused by non-inertial cavitation. It is also worth noting that PUT can produce either non-inertial or inertial cavitation based on the applied energy levels [68]. Previous studies reported that the maximum depth of the PUT treatment was approximately 1 cm [86]. A high laser fluence at the surface or targeted laser delivery using optical fiber is required to achieve greater treatment depths. Further, the cavitation nuclei will also influence the result.

The limitations of the study are the use of the complete medium containing phenol red in place of blood, and the fluid flow was in a straight channel, while the blood vessels are not perfectly straight. The use of phenol red can also offer optical absorption similar to real blood, a material property that is fundamental to PUT [3]. Though the phenol red compensates for the absorption of the blood, it cannot account for the scattering properties of blood. The size of the channel is 450 μm in diameter, which would represent a large vessel because using a vessel diameter smaller than that would result in a smaller number of cells in the channel. The smaller number of cells could affect the sensitivity of the assay to measure the amount of NO and PGI_2_ released from the cells. Moreover, the state of cells post-treatment is not thoroughly evaluated. In the future, the effect of ultrasound and PUT on other biochemical productions related to endothelial cells can be studied using this vessel model.

The other limitation of our study is that the vessel model was produced under a static condition. While the advantage of the static culture is that it does not cause any mechanotransduction associated with shear stress on the endothelial cells during cell culture, the real in vivo situation always involved shear stress [87]. Further study will be needed to investigate the effect of different cell culture conditions. Moreover, the current study focuses on understanding the effects of PUT on the release of NO and PGI_2_. We recognize that there are many other biomarkers related to the endothelial cell damage and dysfunction [88,89]. The characterization of these markers may provide a full picture of endothelial dysfunction, which is, however, out of the scope of the current study.

## 5. Conclusions

In conclusion, the current study demonstrates in an in vitro blood vessel model that while both ultrasound-only and laser-only treatments can increase the release of NO and PGI_2_ at low energy levels, the combination of laser treatment and ultrasound, i.e., PUT, would reduce the level of increase in NO and PGI_2_ at similar laser and ultrasound energy levels. This reduction was likely related to the dysfunction of endothelial cells due to the enhanced cavitation activities during PUT. The findings reported in this study provide a possible mechanism to explain the anti-vascular effect of PUT.

## 6. Patents

The authors X.Y., X.W. and Y.P. are inventors on a University of Kansas patent application, “Method and Apparatus for removing microvessels”, related to this manuscript and technology.

## Supplementary Material

Suppl. Figures

## Data Availability

The data supporting reported results are available from the corresponding author, X.Y., upon reasonable request.
