# Supplementary material for: Effect of Photo-Mediated Ultrasound Therapy on Nitric Oxide and Prostacyclin from Endothelial Cells
Source: Appl Sci (Basel). Author manuscript; Available in PMC 2022 Aug 17. (PMC9384428; doi:10.3390/app12052617)
Supplement: Suppl. Figures [file NIHMS1829631-supplement-Suppl__Figures.docx]

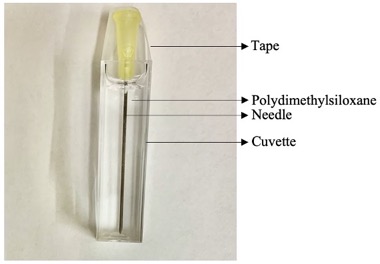


Figure S1. A picture of the fabrication of blood vessel microchannel


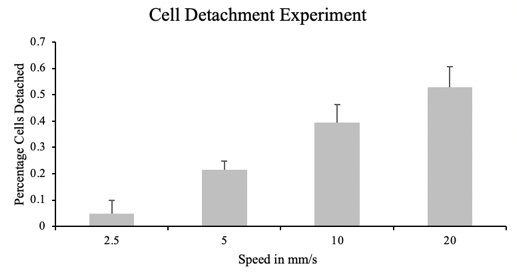


**Figure S2.** Percentage of cells detached at different speeds 2.5, 5, 10 and 20 mm/s. The cellular detachment was least at 2.5 mm/s.

| **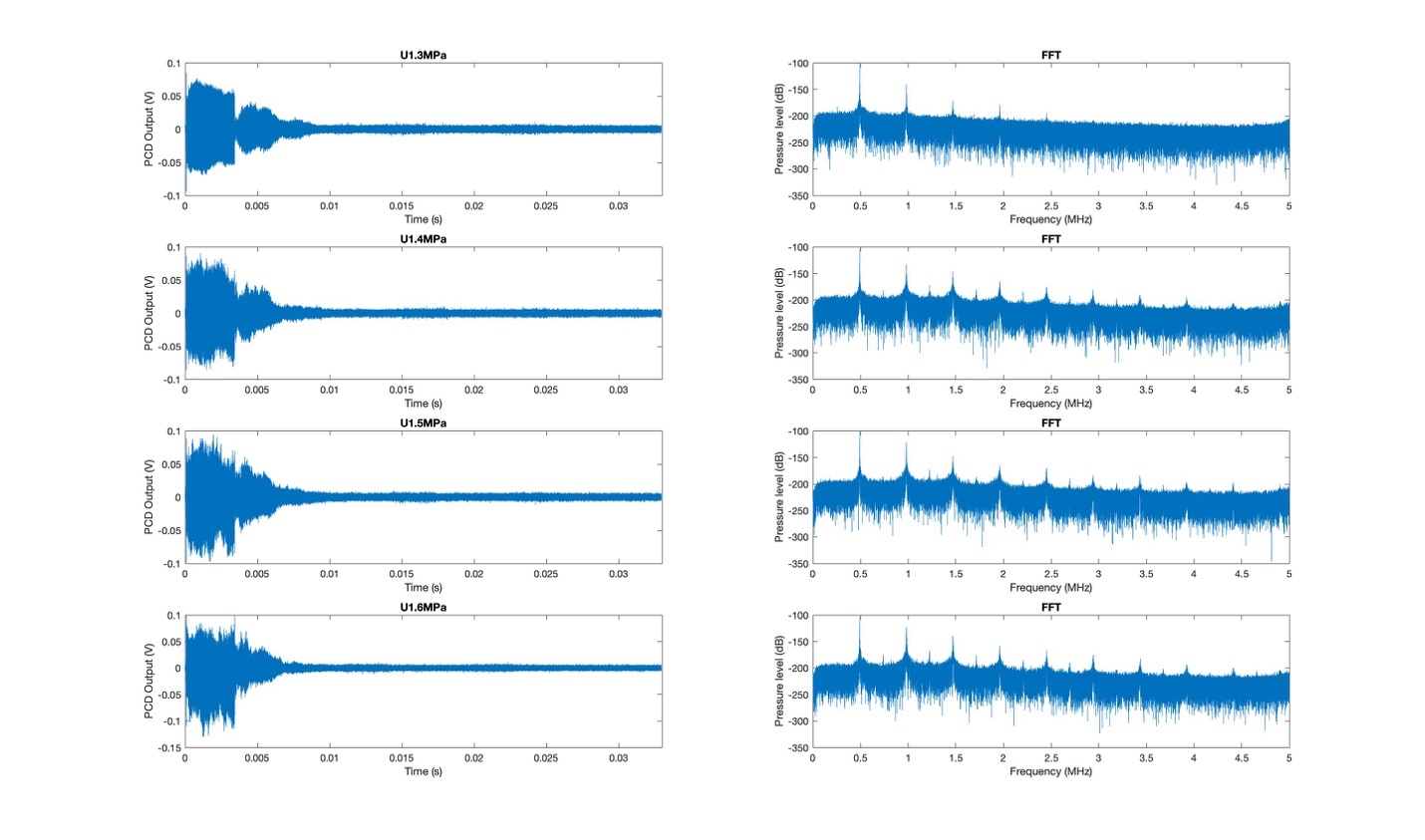** | **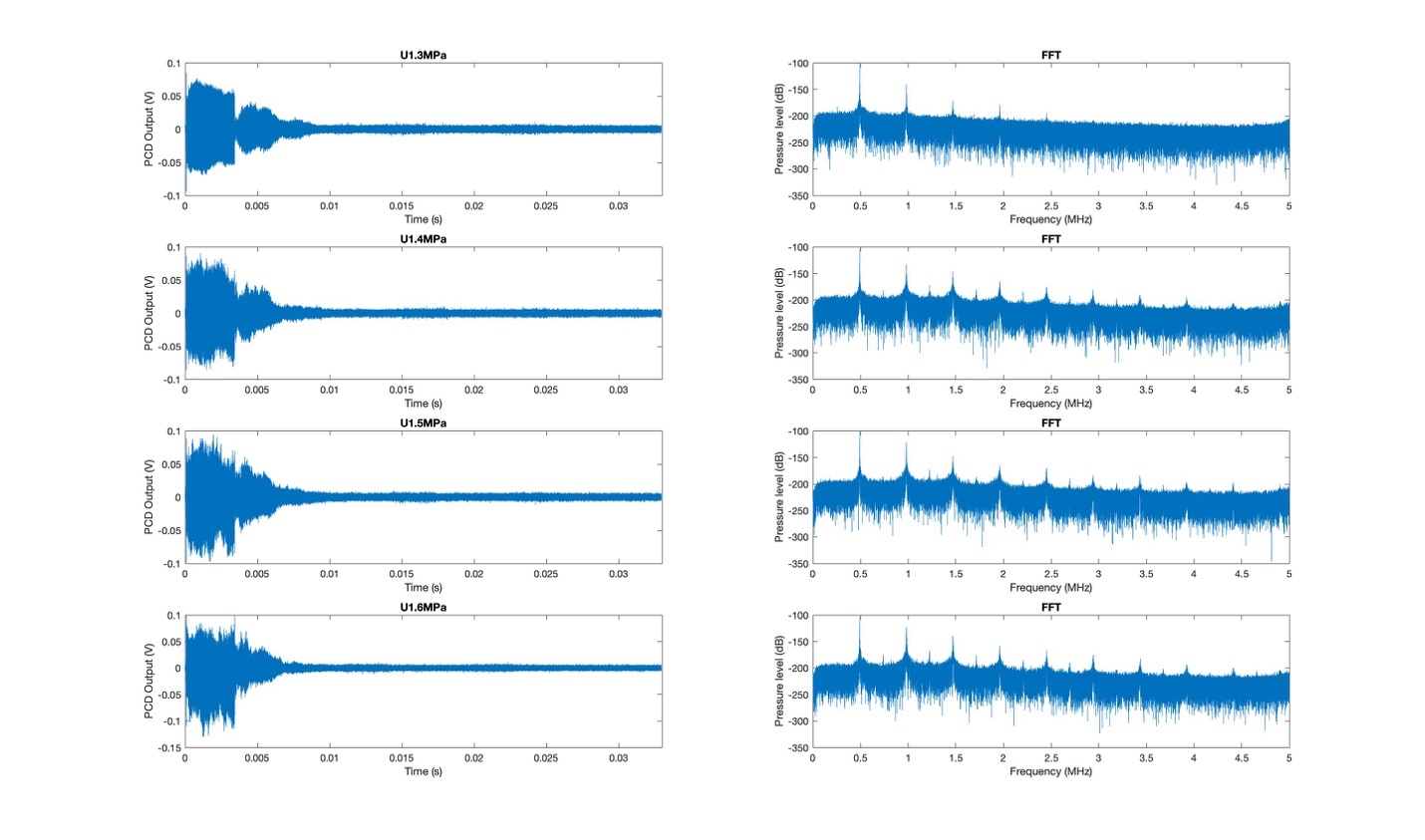** |
| --- | --- |
| **(a)** | **(b)** |
| **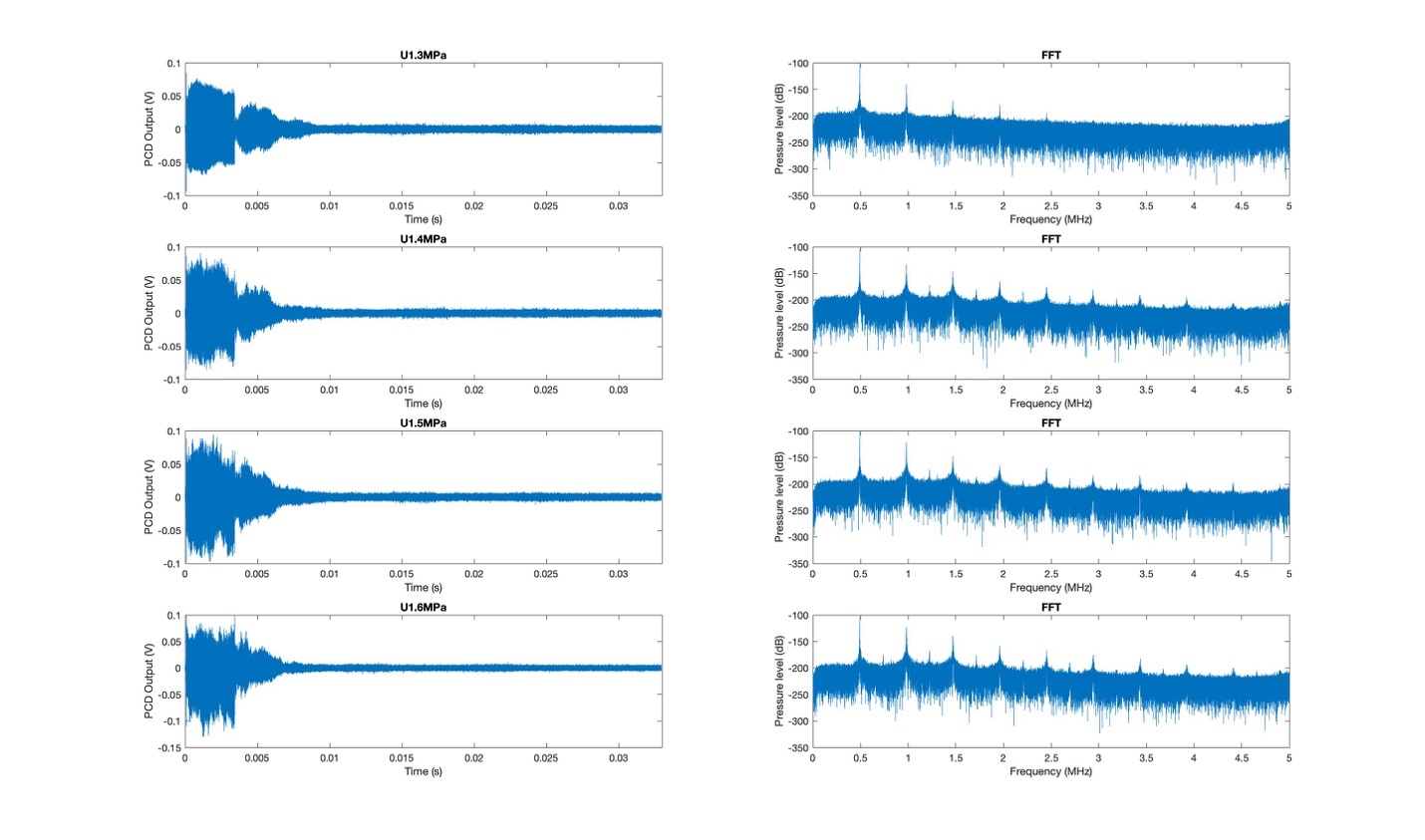** | **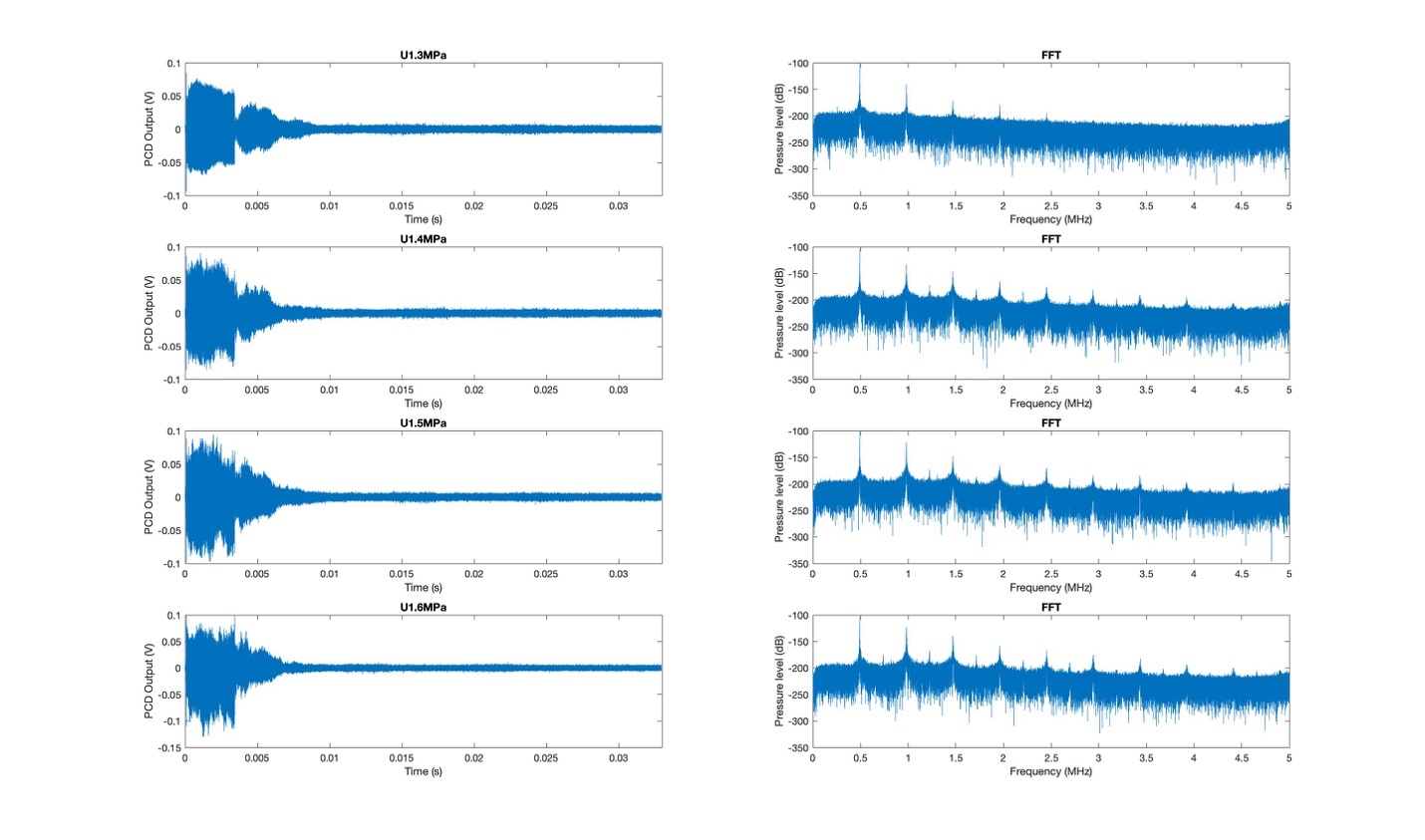** |
| **(c)** | **(d)** |

**Figure S3.** FFT analysis of the detected cavitation signals for ultrasound only. Signals at (a) 1.3MPa and (b) 1.4MPa and, their corresponding FFTs (c) and (d).

| **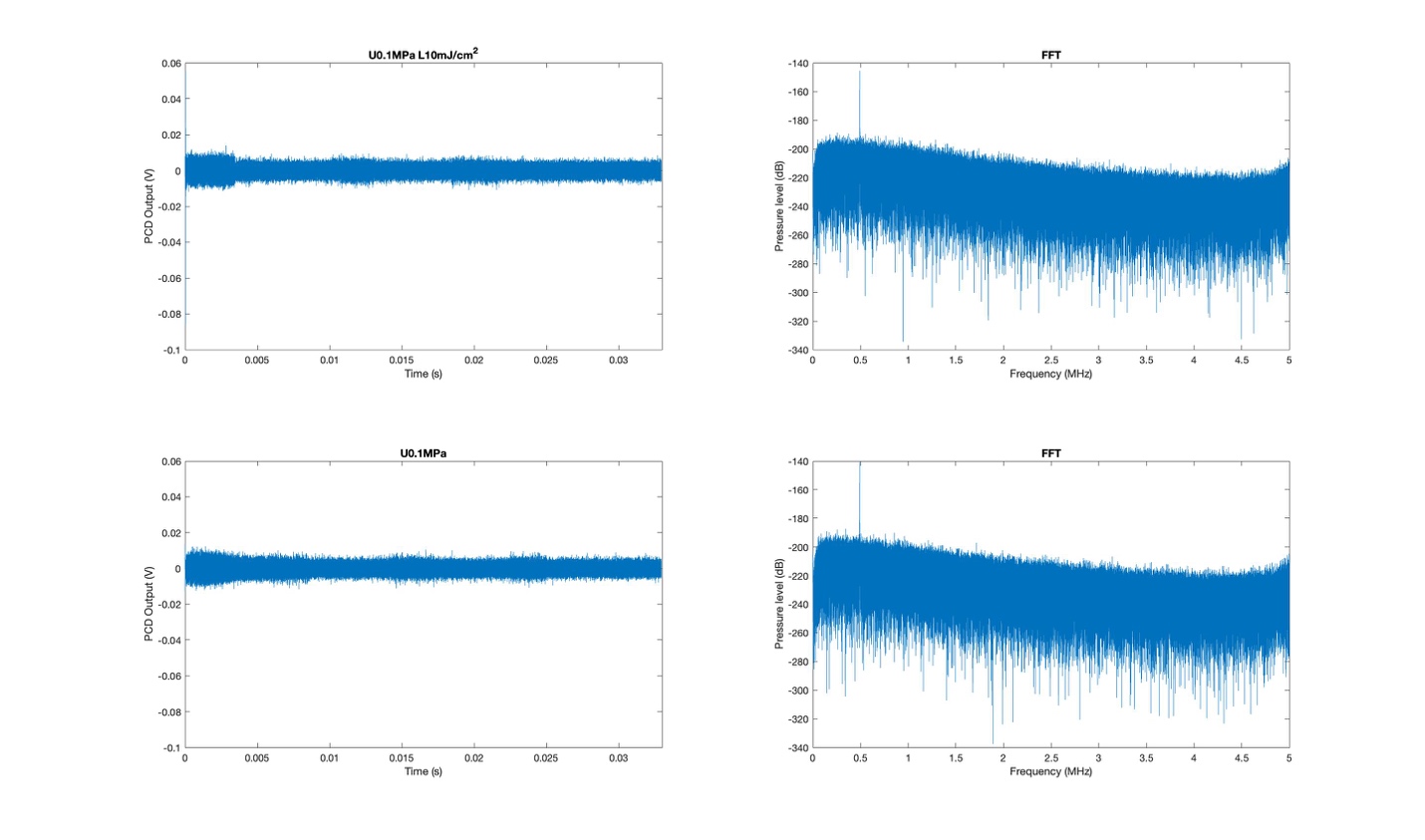** | **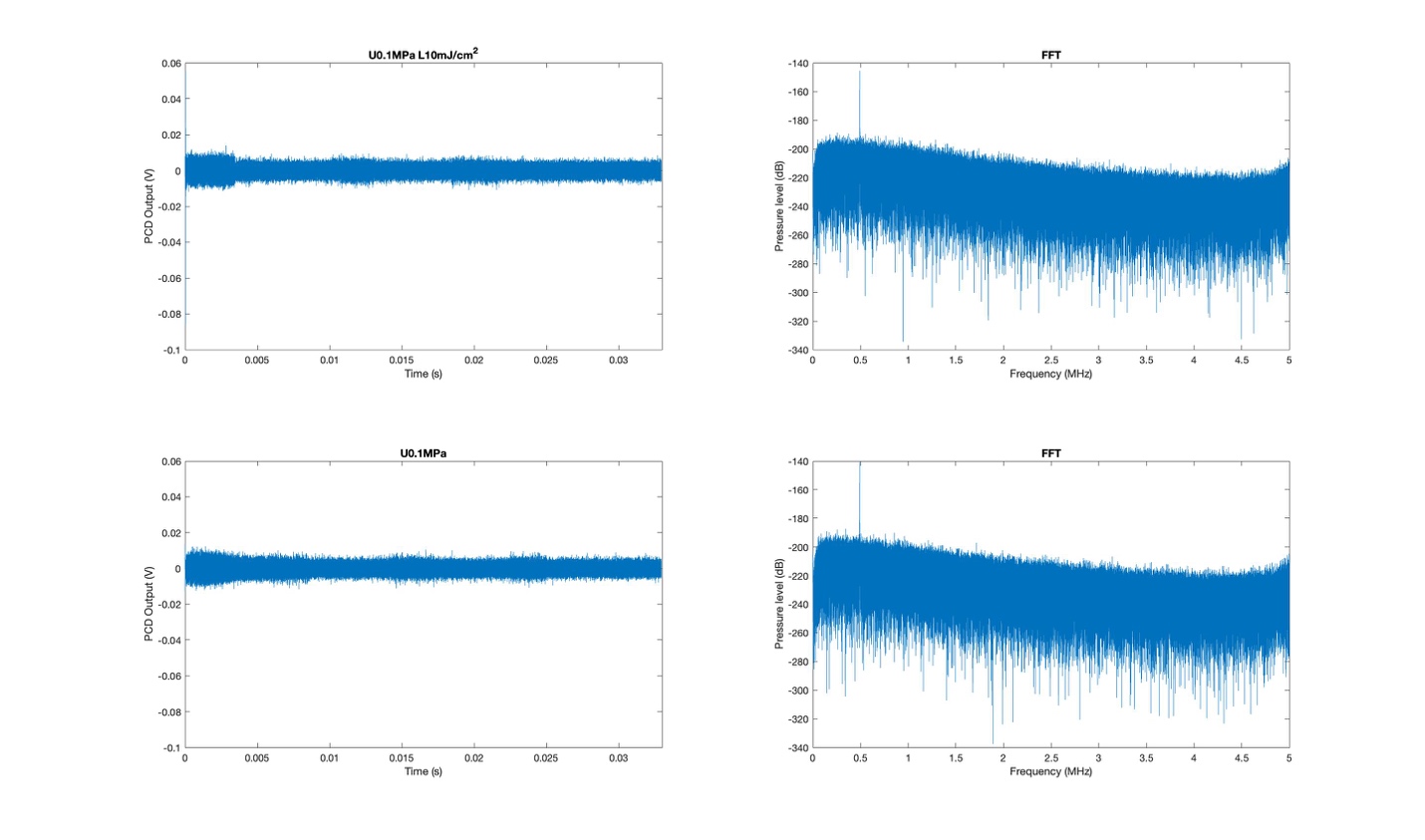** |
| --- | --- |
| **(a)** | **(b)** |
| **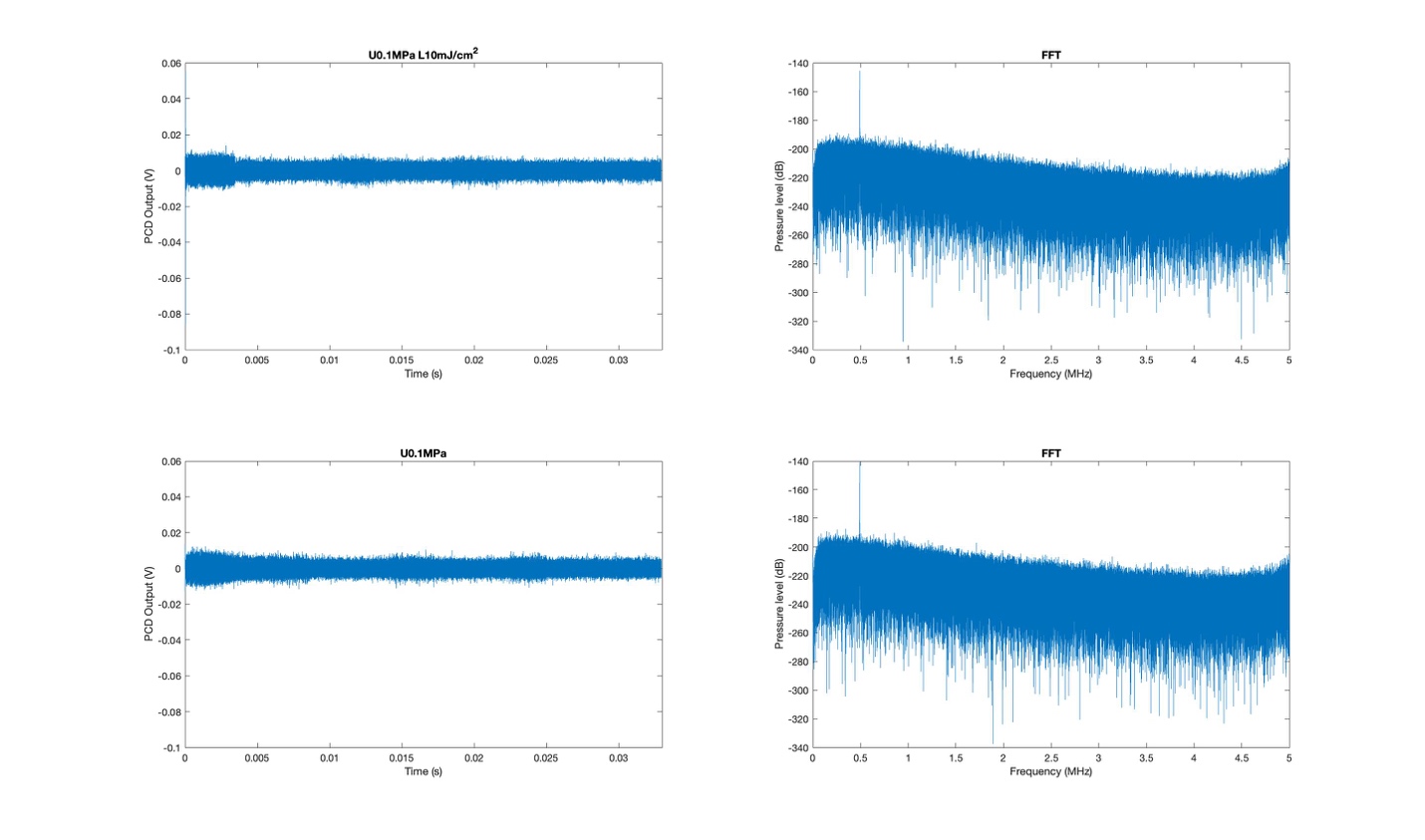** | **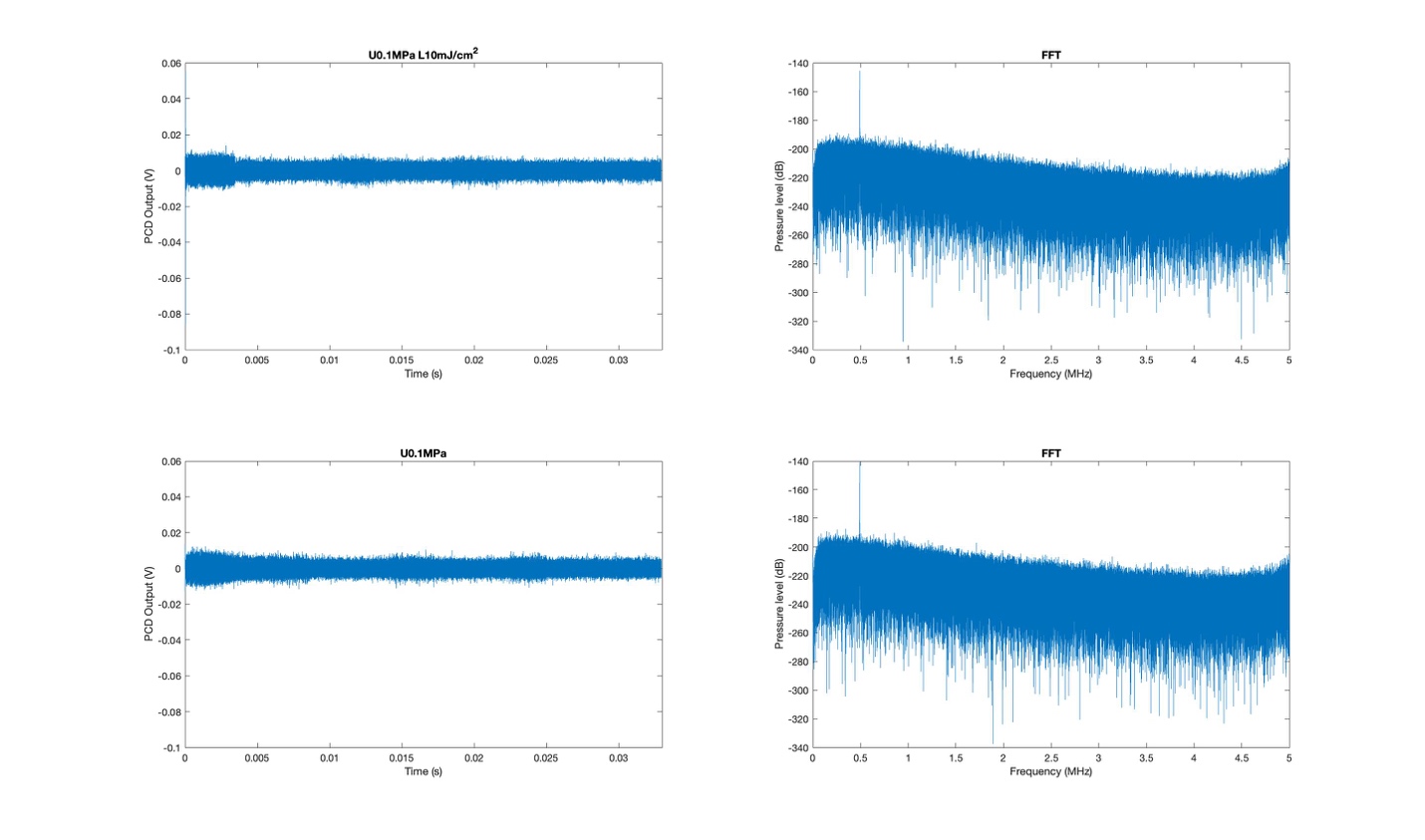** |
| **(c)** | **(d)** |

**Figure S4.** FFT analysis of the detected cavitation signals for (a) 0.1MPa ultrasound only and 0.1MPa ultrasound applied synchronously with 10mJ/cm^2^ laser and, their corresponding FFTs (c) and (d).

| **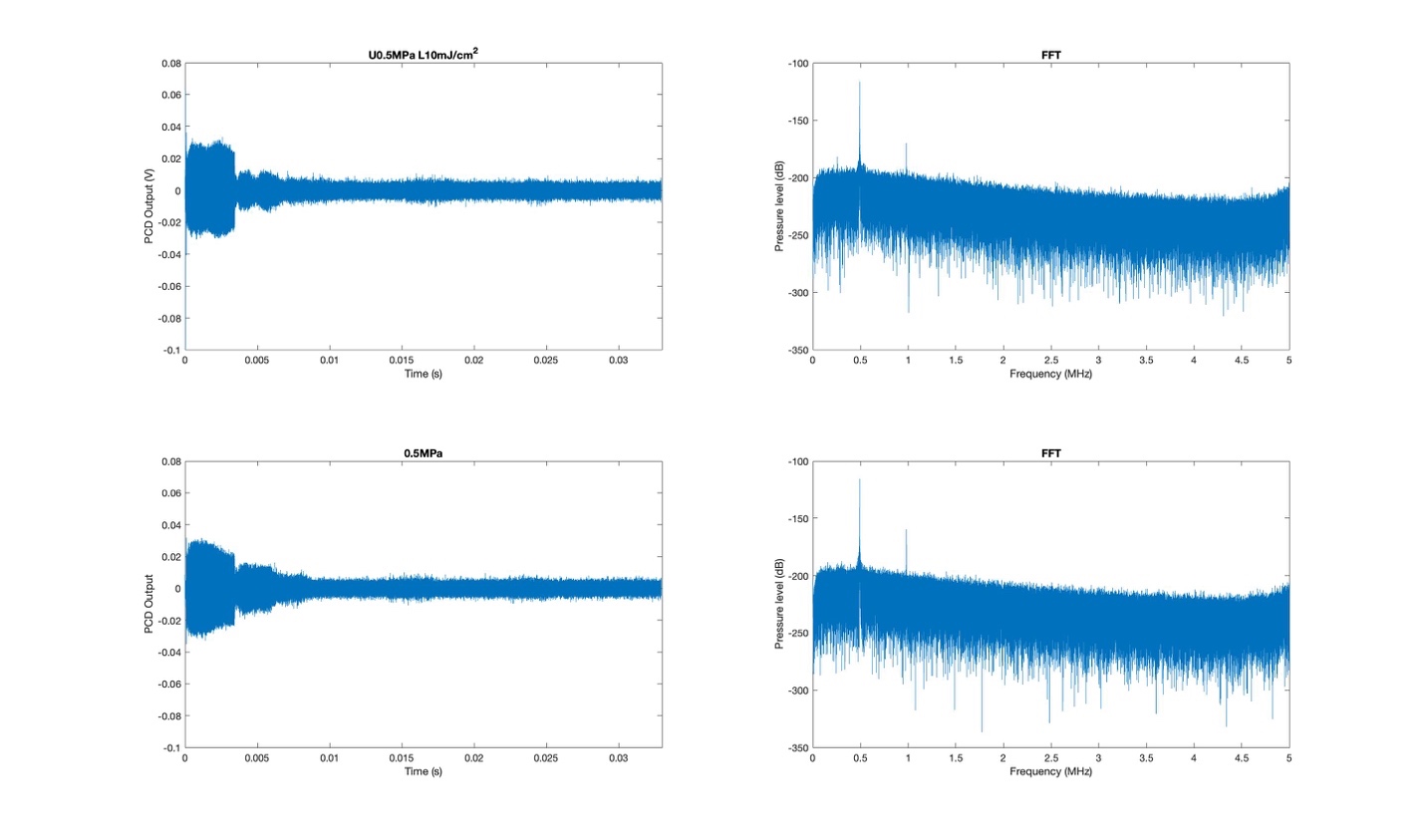** | **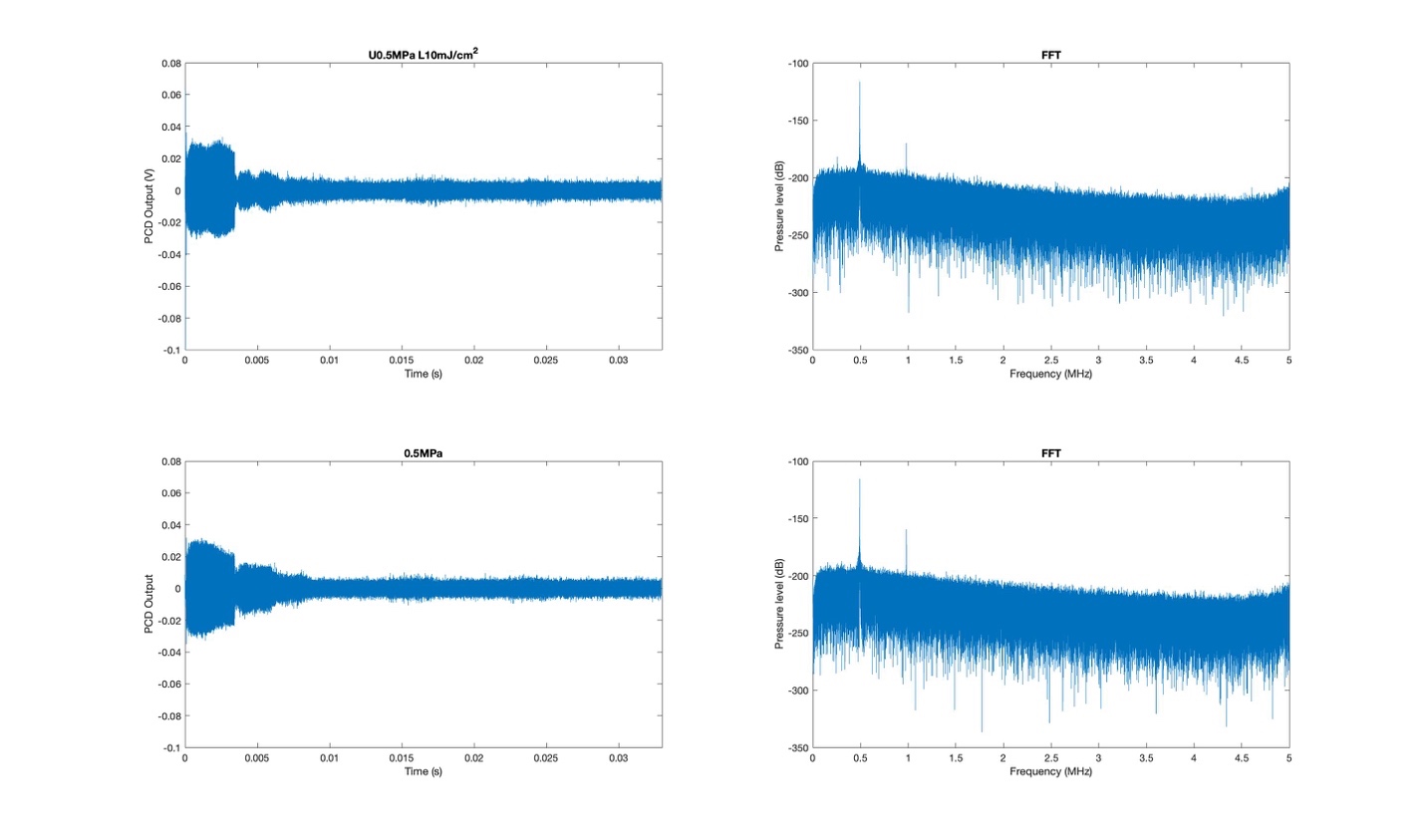** |
| --- | --- |
| **(a)** | **(b)** |
| **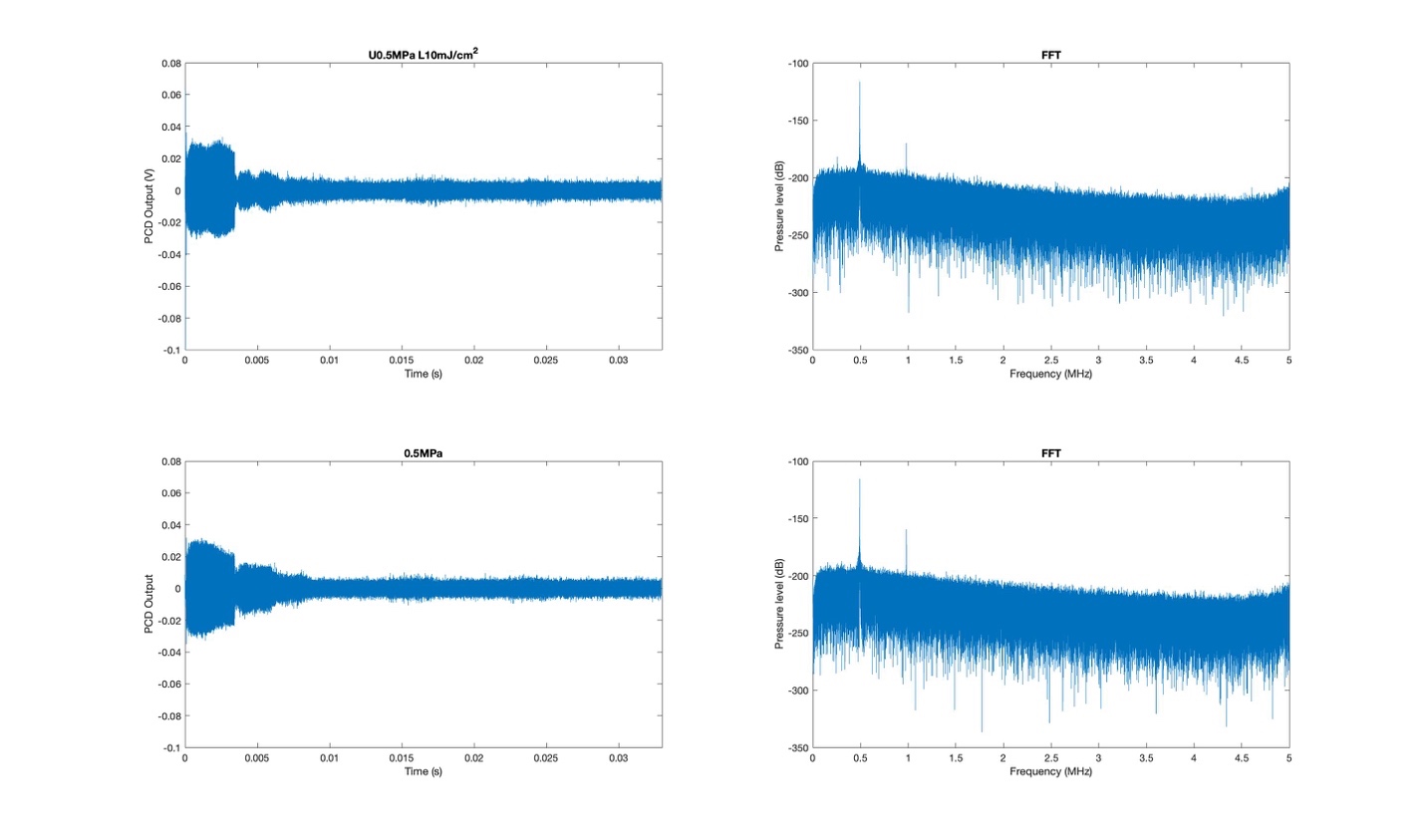** | **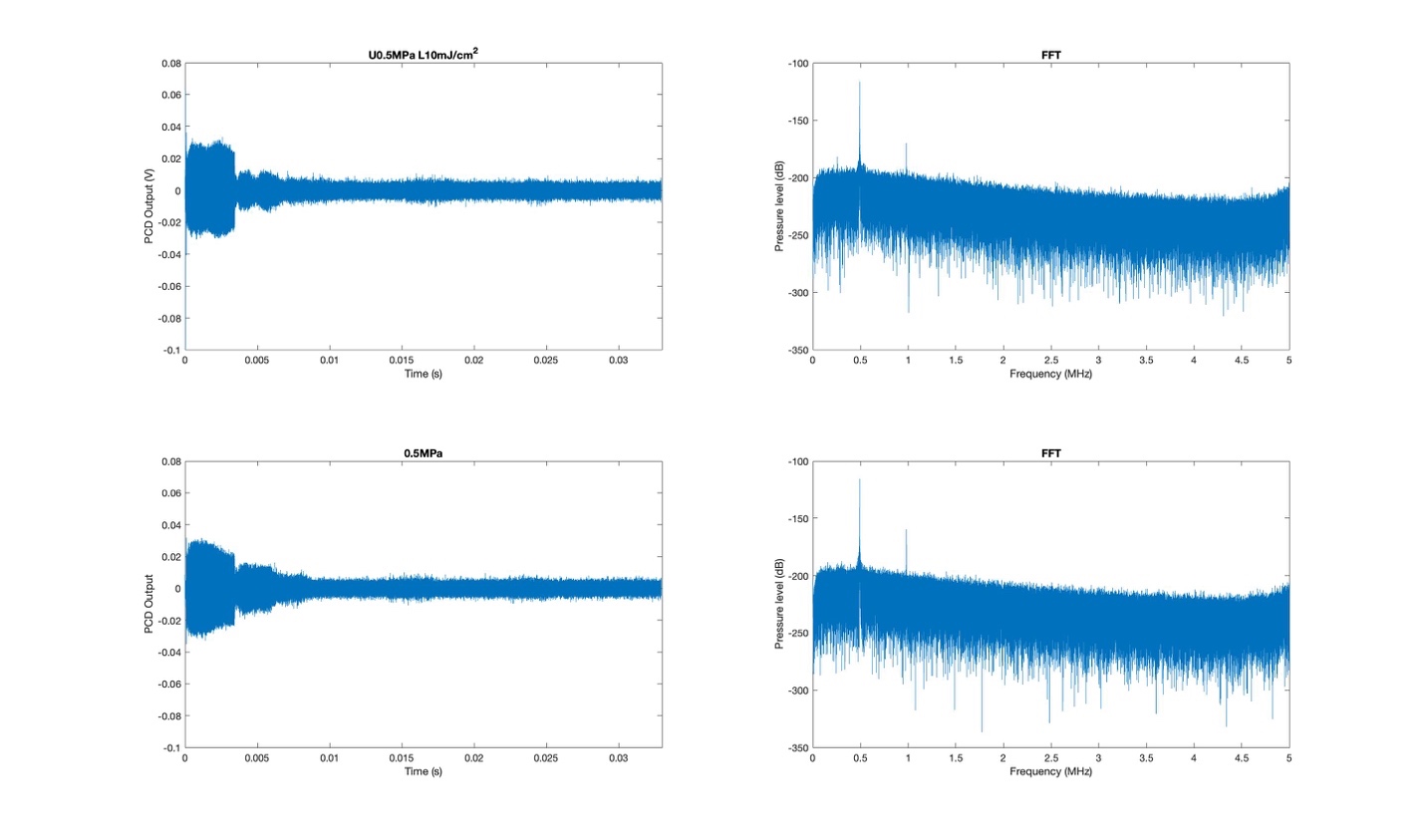** |
| **(c)** | **(d)** |

**Figure S5.** FFT analysis of the detected cavitation signals for (a) 0.5MPa ultrasound only and 0.5MPa ultrasound applied synchronously with 10mJ/cm^2^ laser and, their corresponding FFTs (c) and (d).

| **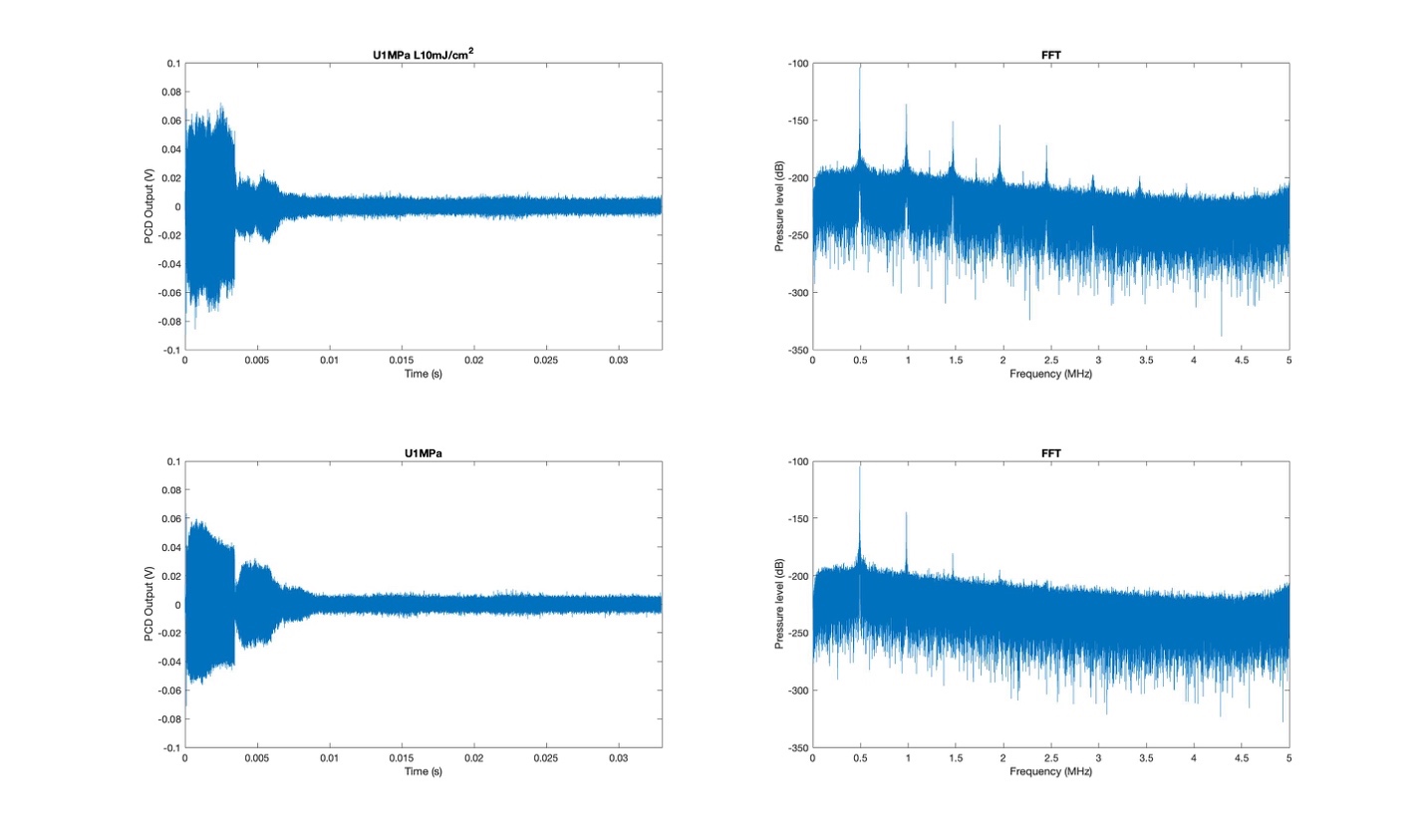** | **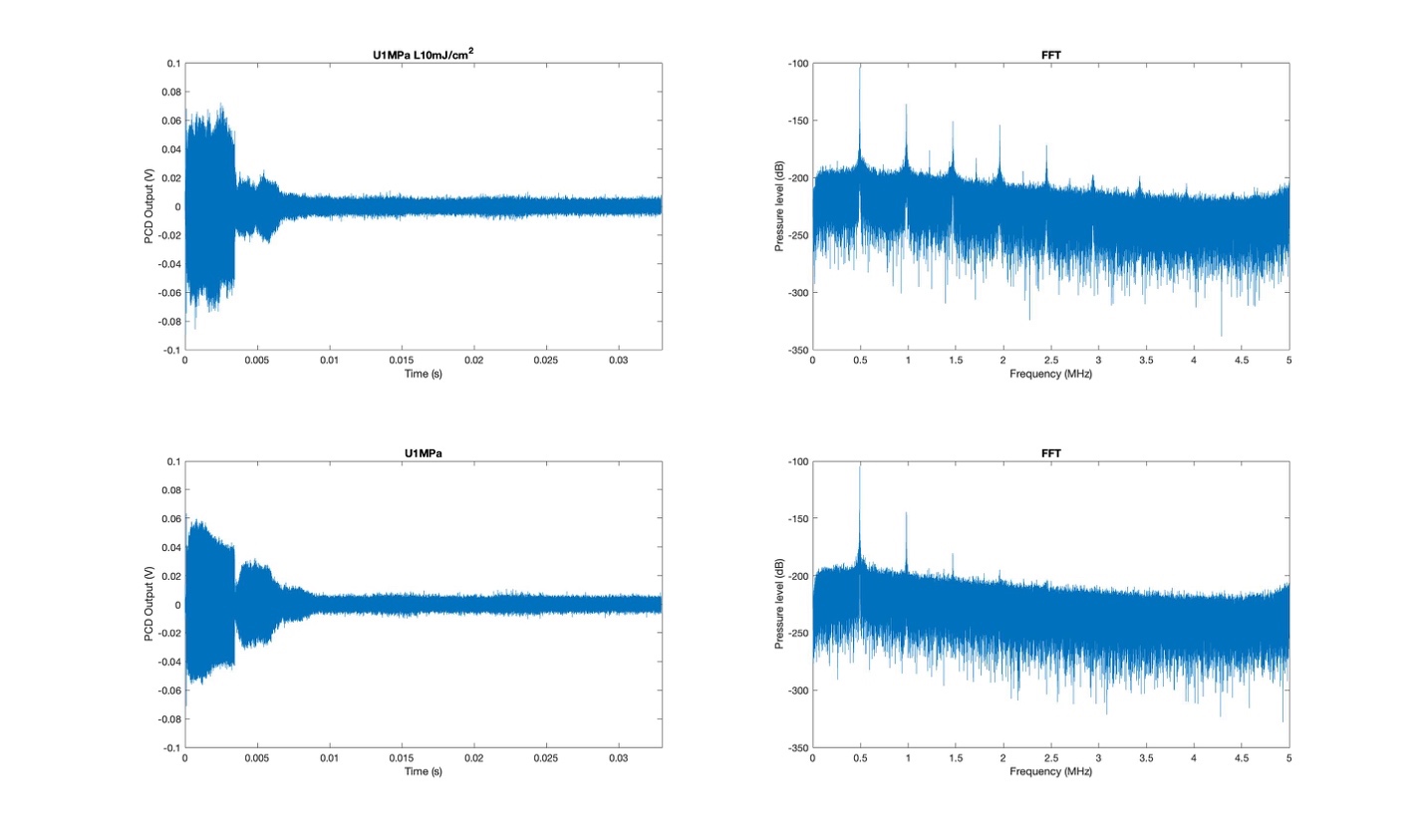** |
| --- | --- |
| **(a)** | **(b)** |
| **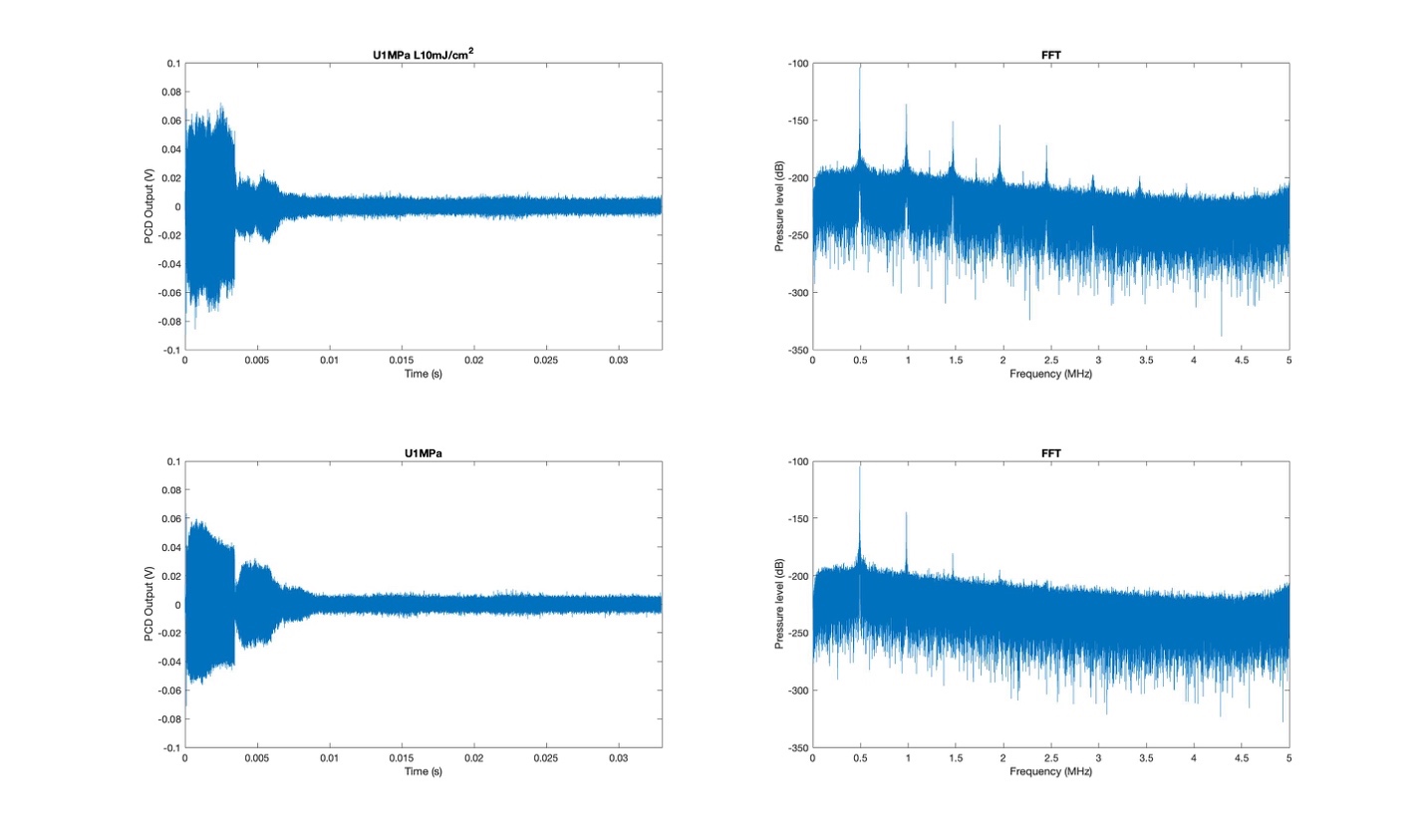** | **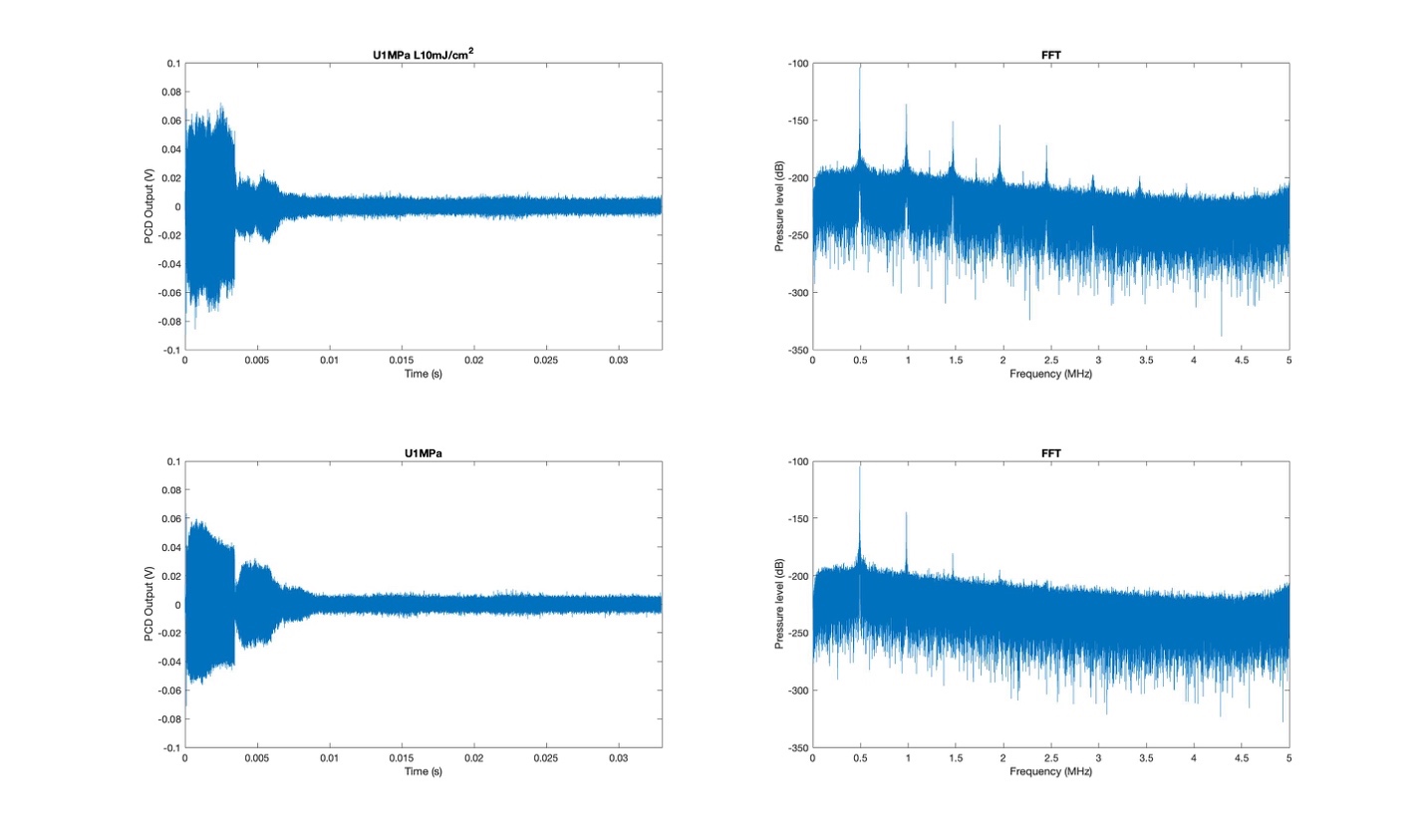** |
| **(c)** | **(d)** |

**Figure S6.** FFT analysis of the detected cavitation signals for (a) 1MPa ultrasound only and 1MPa ultrasound applied synchronously with 10mJ/cm^2^ laser and, their corresponding FFTs (c) and (d).

| **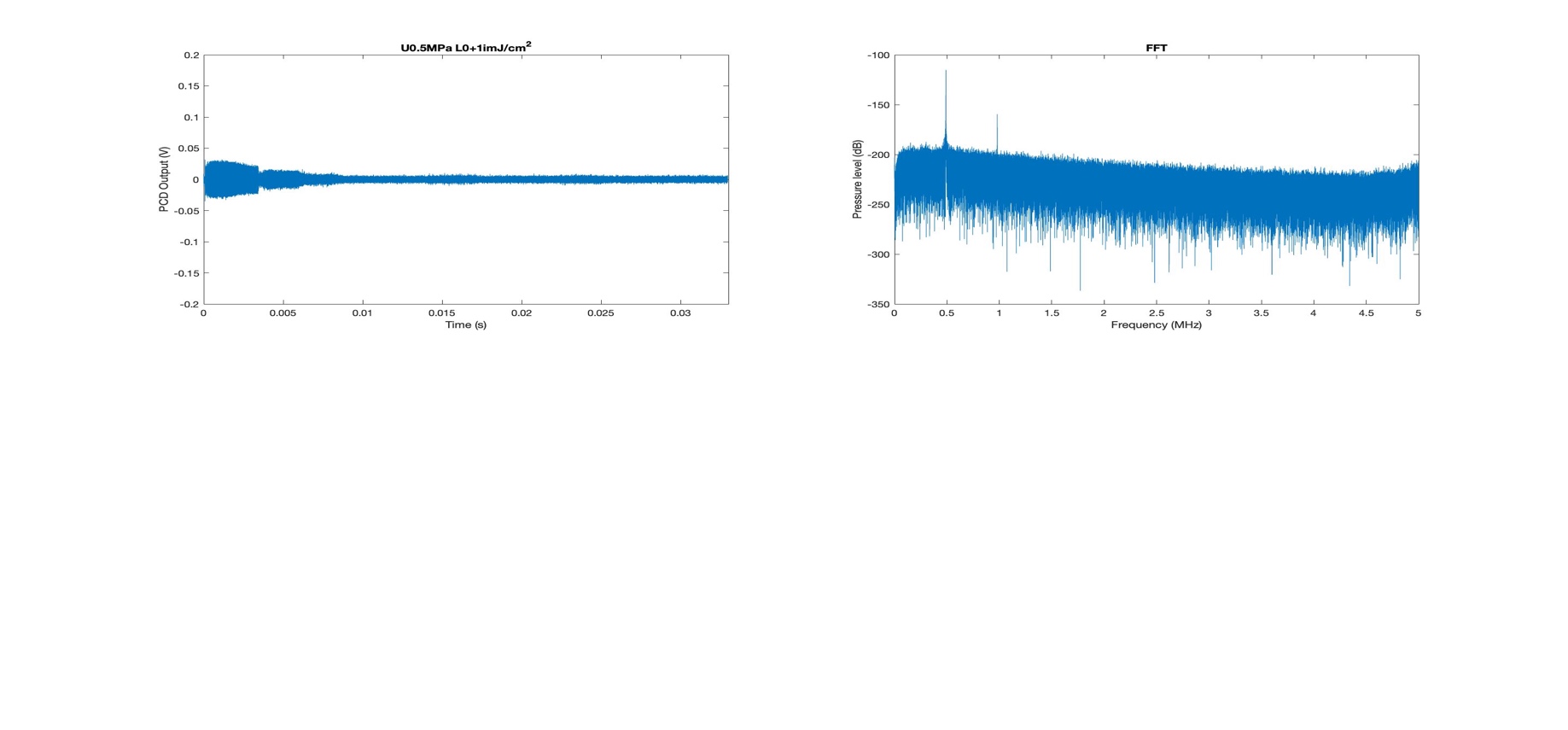** | **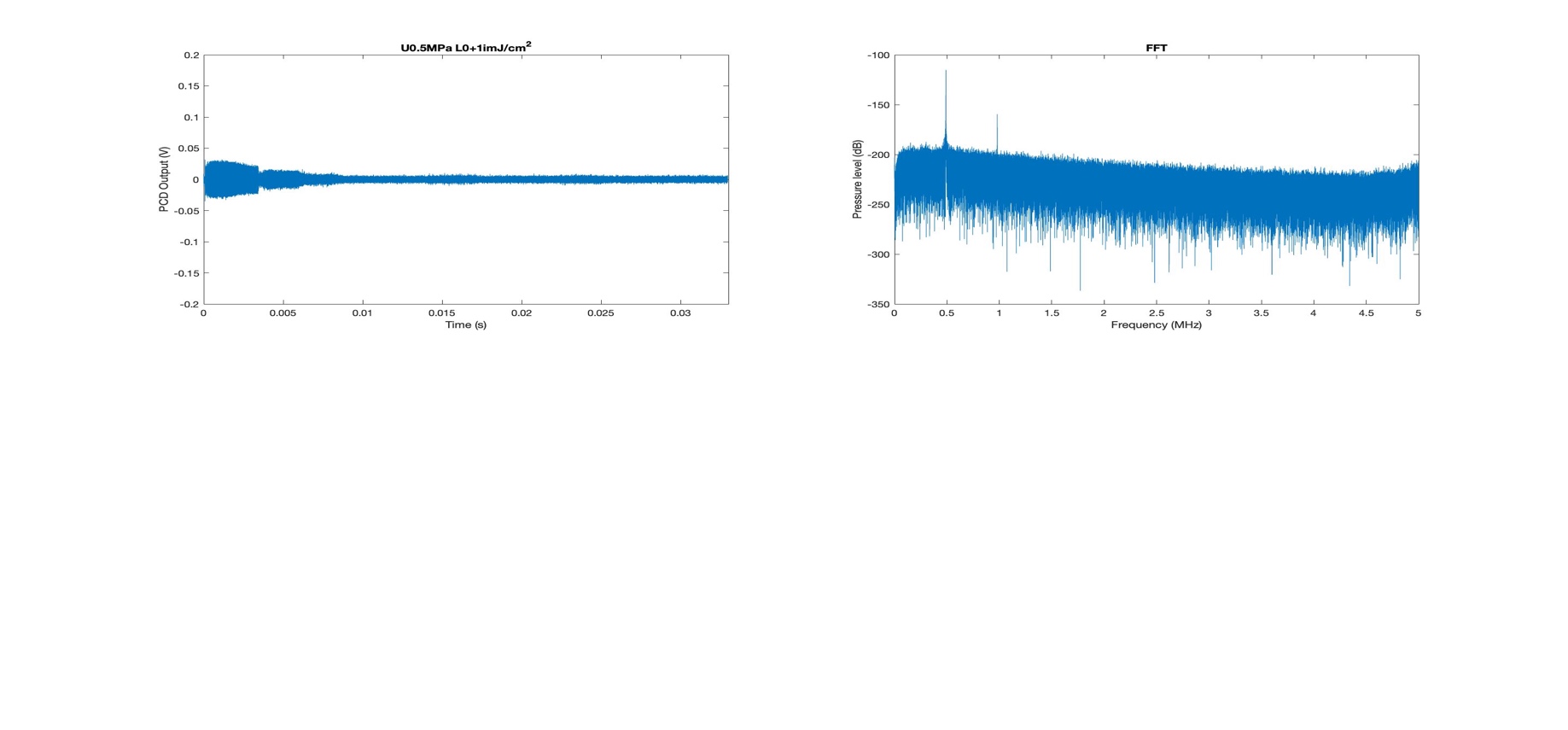** |
| --- | --- |
| **(a)** | **(b)** |
| **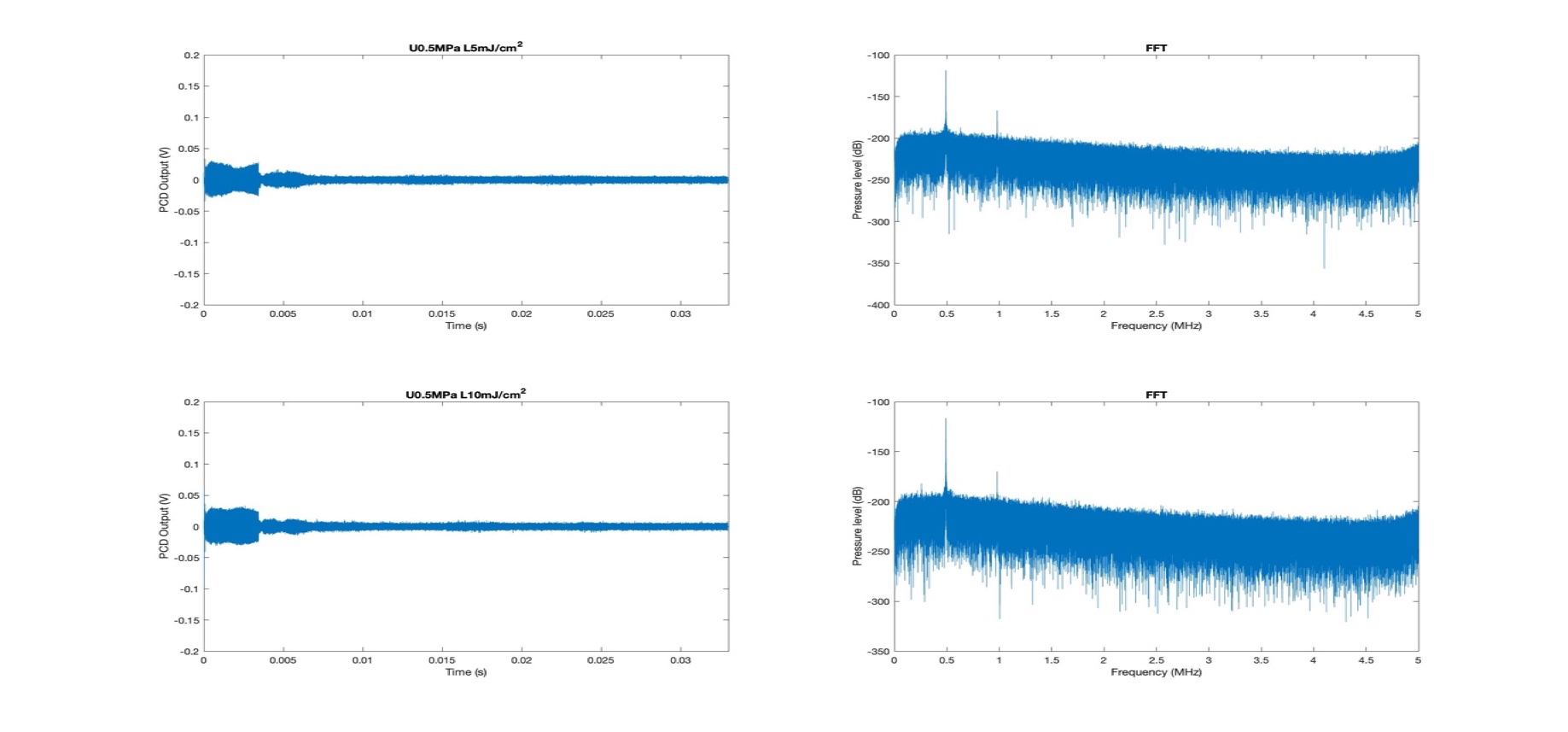** | **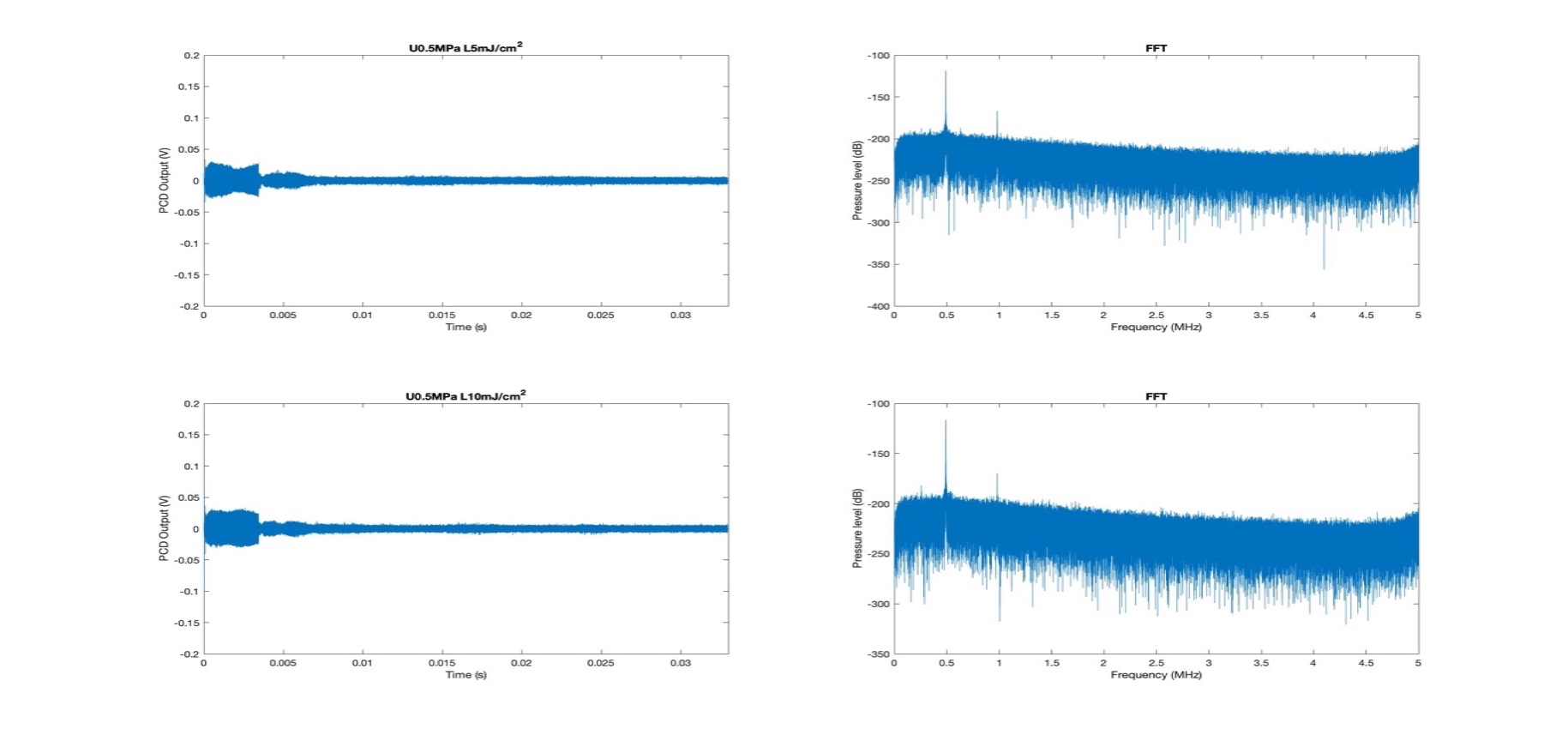** |
| **(c)** | **(d)** |
| **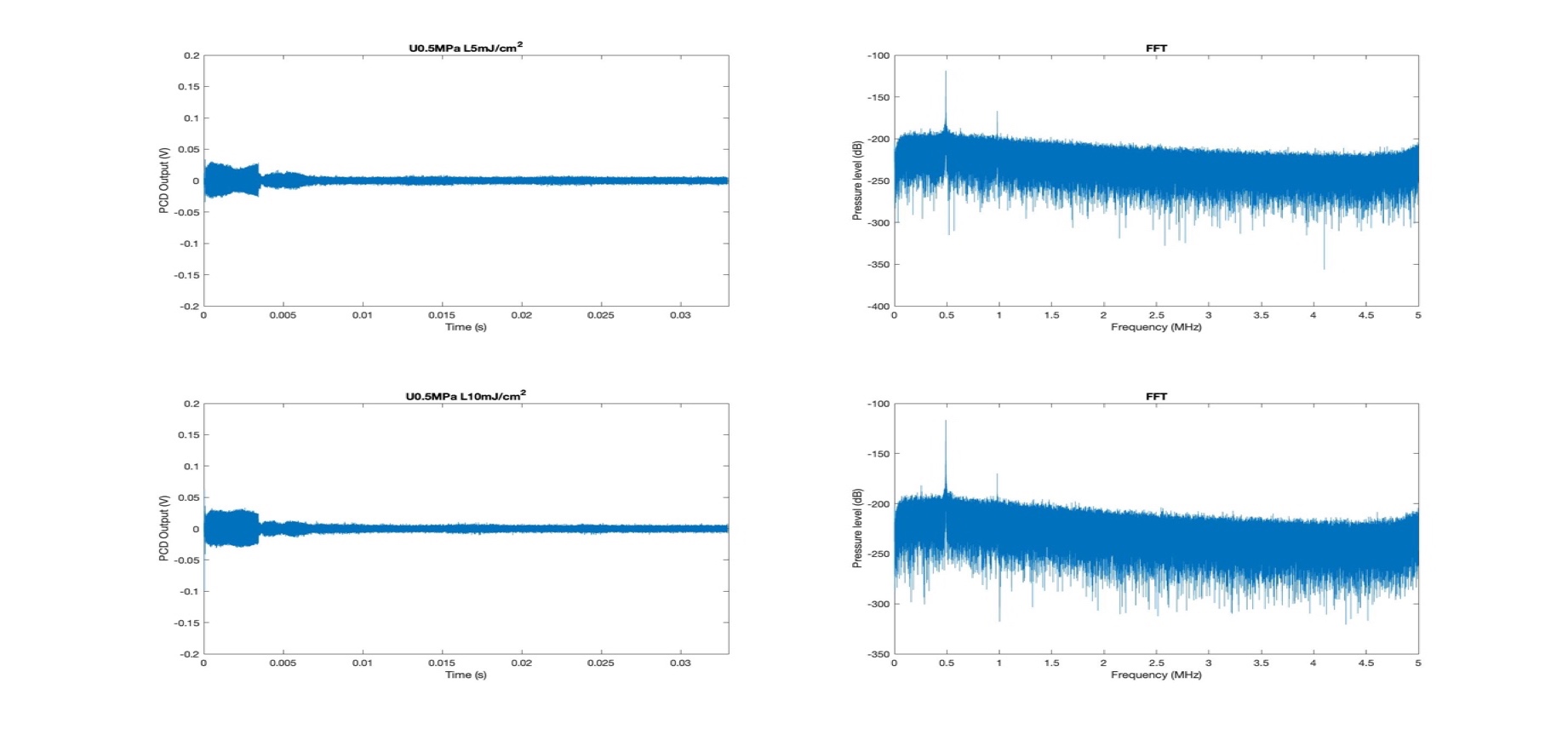** | **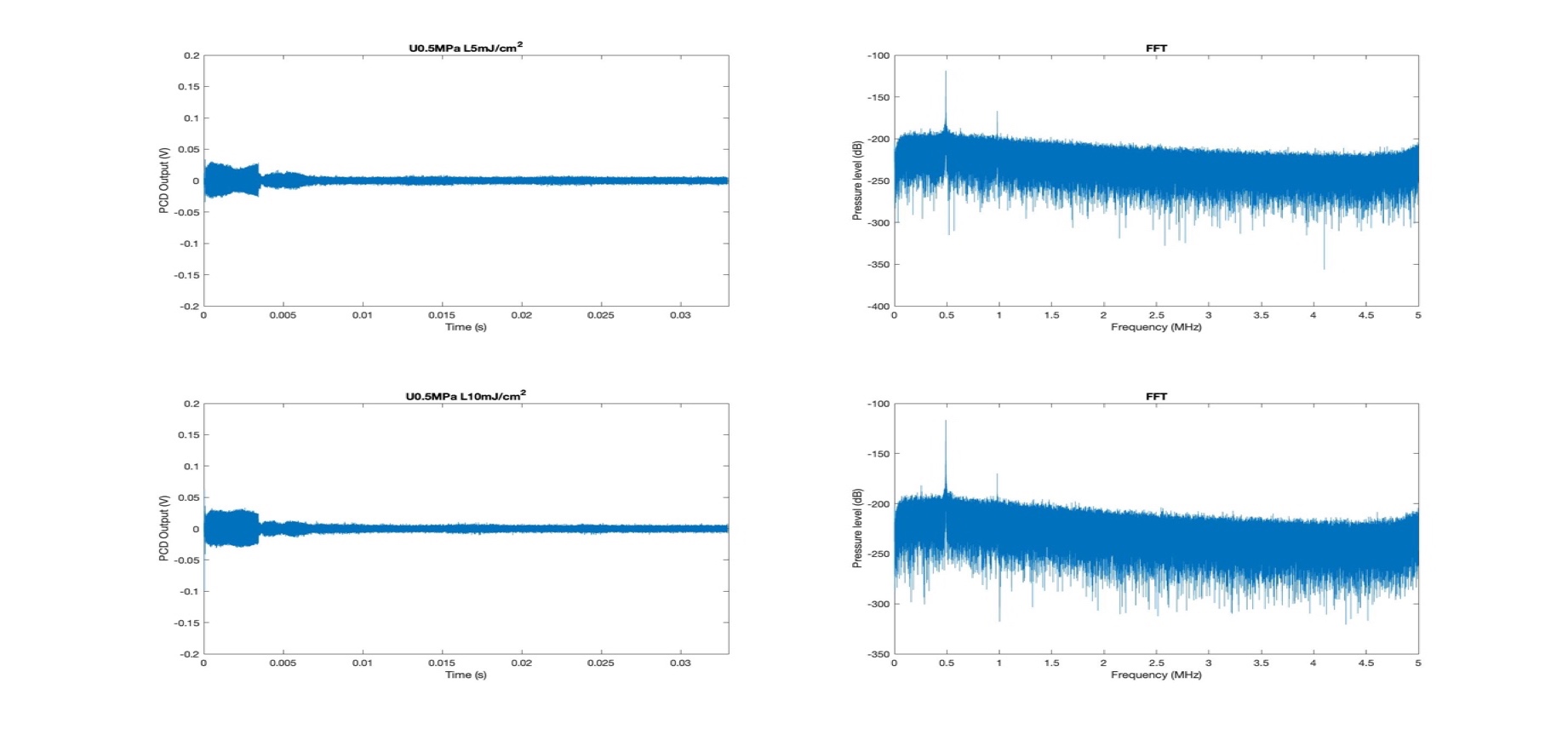** |
| **(e)** | **(f)** |
| **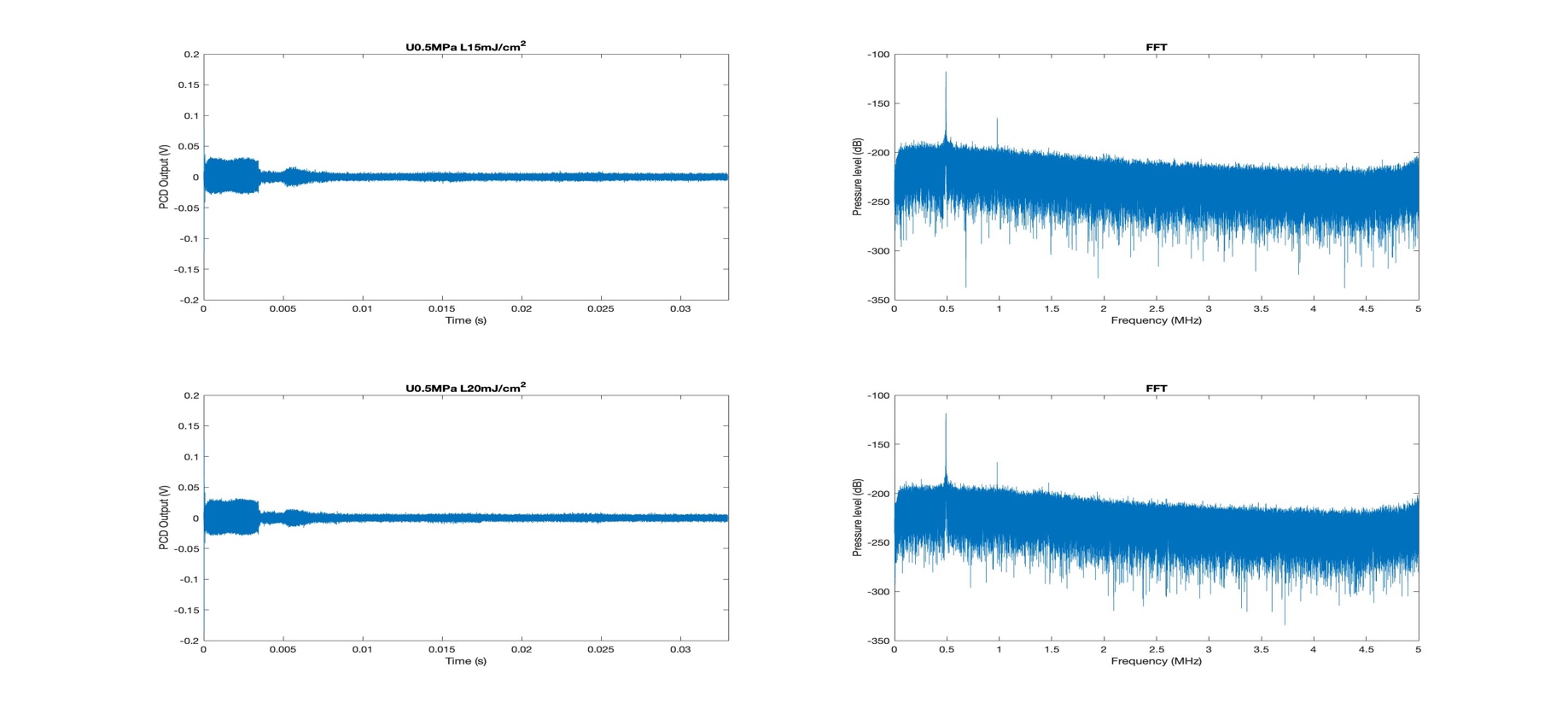** | **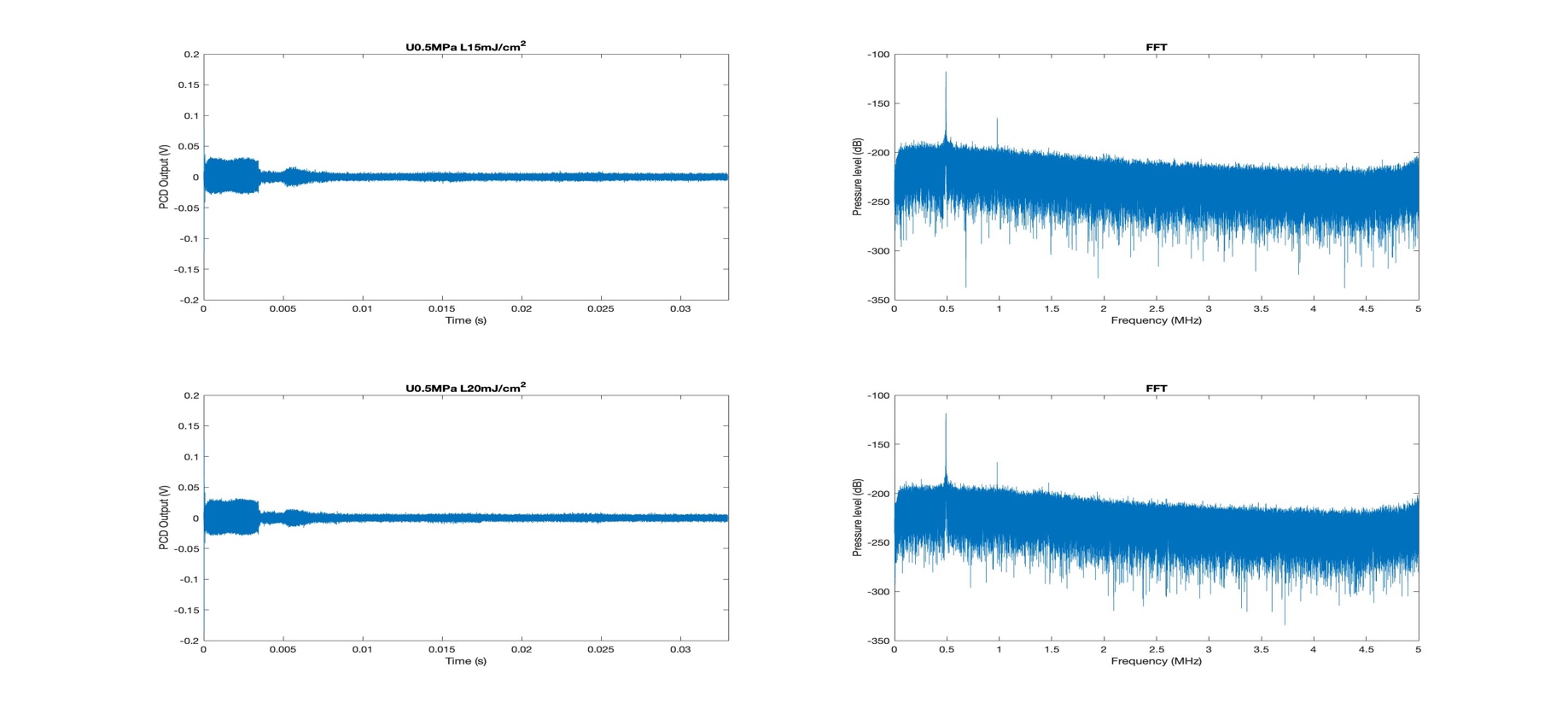** |
| **(g)** | **(h)** |
| **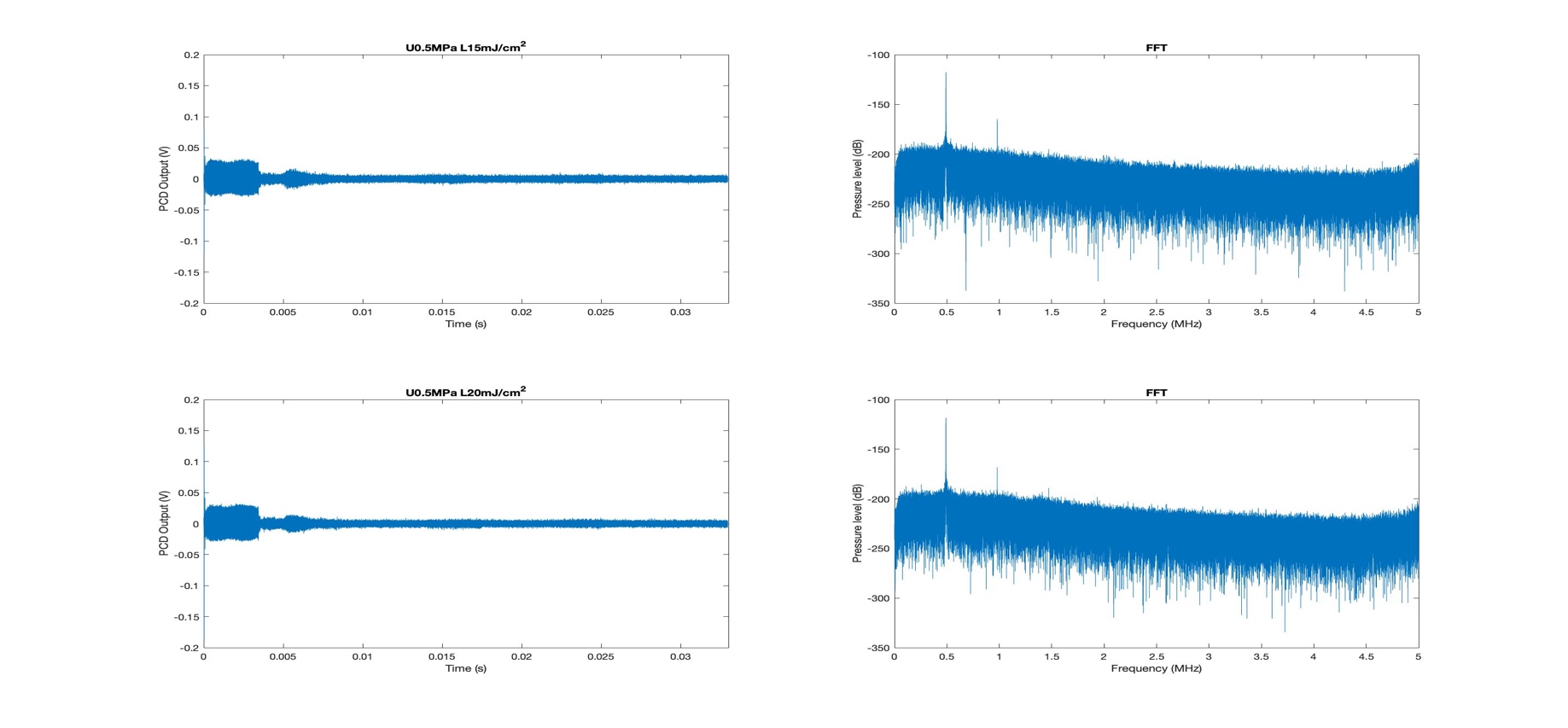** | **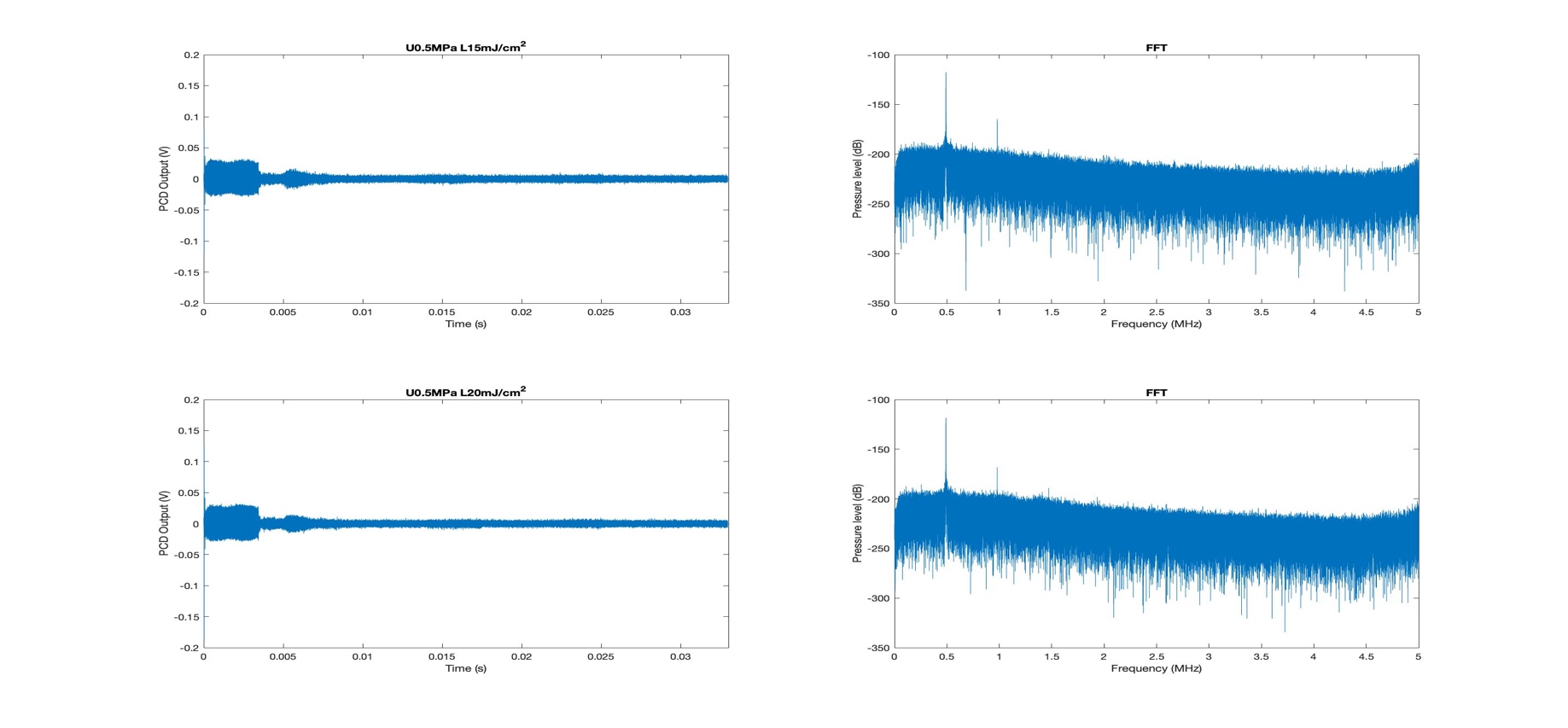** |
| **(i)** | **(j)** |

**Figure S7.** FFT analysis of the detected cavitation signals for (a) ultrasound only and ultrasound peak negative pressure of 0.5MPa applied synchronously with varying laser fluences (b)5, (c)10, (d)15 and (e)20 mJ/cm^2^. The corresponding FFTs are shown in (g-j).
